# Applications of genetic code expansion technology in eukaryotes

**DOI:** 10.1093/procel/pwad051

**Published:** 2023-10-17

**Authors:** Qiao-ru Guo, Yu J Cao

**Affiliations:** State Key Laboratory of Chemical Oncogenomic, Guangdong Provincial Key Laboratory of Chemical Genomics, Peking University Shenzhen Graduate School, Shenzhen 518055, China; State Key Laboratory of Chemical Oncogenomic, Guangdong Provincial Key Laboratory of Chemical Genomics, Peking University Shenzhen Graduate School, Shenzhen 518055, China; Institute of Chemical Biology, Shenzhen Bay Laboratory, Shenzhen 518132, China

**Keywords:** genetic code expansion, unnatural amino acid, eukaryotes, basic research, therapeutic applications

## Abstract

Unnatural amino acids (UAAs) have gained significant attention in protein engineering and drug development owing to their ability to introduce new chemical functionalities to proteins. In eukaryotes, genetic code expansion (GCE) enables the incorporation of UAAs and facilitates posttranscriptional modification (PTM), which is not feasible in prokaryotic systems. GCE is also a powerful tool for cell or animal imaging, the monitoring of protein interactions in target cells, drug development, and switch regulation. Therefore, there is keen interest in utilizing GCE in eukaryotic systems. This review provides an overview of the application of GCE in eukaryotic systems and discusses current challenges that need to be addressed.

## Introduction

Genetic code expansion (GCE) has gained considerable interest in protein engineering, as it allows the site-specific incorporation of unnatural amino acids (UAAs) into proteins, resulting in the introduction of novel properties and functions. Proteins are composed of 20 natural amino acids, which display a wide range of functions. The limited number of building blocks constrains the ability to modify their function and explore their structure and interactions. The use of UAAs through GCE offers a promising avenue for expanding the diversity and functionality of proteins.

To overcome the limitations of the 20 natural amino acids, modified UAAs have been introduced to alter the physicochemical and biological properties of peptides and proteins. UAAs were first reported to be incorporated into dipeptides in 1983 (Heckler et al., 1983) and subsequently, in 1989, they were introduced into proteins (Noren et al., 1989). Since then, the application of GCE technology has steadily expanded to include enzyme engineering, drug discovery, conditional control of biological processes, biophysical probing, and posttranscriptional modification (PTM), among others, by utilizing UAAs incorporated into multiple proteins to meet diverse research needs. At present, most GCE techniques are conducted primarily in prokaryotic systems such as *Escherichia coli* due to their advantages of cost-efficiency, high yield, and ease of operation. However, there is a growing need to extend these techniques to eukaryotic systems because prokaryotic systems are incapable of introducing PTMs to proteins and thus cannot fully replicate the original functions of proteins in eukaryotes. Moreover, restricting the introduction of UAA exclusively to prokaryotic systems would severely limit the application of GCE in critical areas such as live imaging in eukaryotes and the development of novel eukaryotic cell-based therapeutic strategies.

Since the successful incorporation of UAAs into the genetic code of *Saccharomyces cerevisiae* by Chin et al. in 2003 (Chin et al., 2003), UAA use has gradually been adopted in eukaryotic cells. Compared to prokaryotic expression systems, eukaryotic expression systems can produce more complex glycosylated proteins, including full-length antibodies (Tian et al., 2014). The expansion of the genetic code in eukaryotic cells has greatly broadened the scope of UAA applications, including novel antibody conjugates, vaccines, living cell imaging (Peng and Hang, 2016), and other areas. In the following sections, we will provide a detailed overview of the application of GCE technology in eukaryotic platforms and explore the implications of this technology in eukaryotic systems.

## Eukaryotic platform for gene expression systems

**Figure 1.**
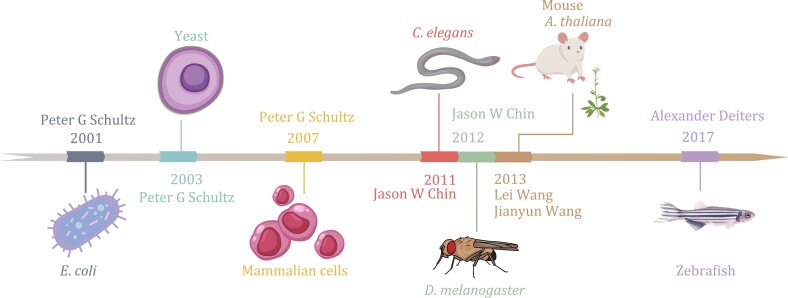
Timeline of the development of genetic code expansion in various organisms.

The first successful incorporation of UAAs into a protein was achieved in *E*. *coli* in 2001 (Wang et al., 2001). Since then, *E*. *coli* has become the most common host organism for GCE because of its simplicity and efficiency (Smolskaya and Andreev, 2019). However, as technology advances and the needs of scientific research evolve, there is an increasing demand for GCE in eukaryotic cells and animals. Over the past few decades, researchers have made significant progress in applying GCE in eukaryotes, such as yeasts, mammalian cells, stem cells, invertebrates, and vertebrates, as host organisms, enabling the resolution of various scientific problems (Fig. 1).

### Technical elements

#### Unnatural amino acids

UAAs are typically chemically synthesized or derived from secondary metabolites of bacteria, fungi, plants, or marine organisms (Blaskovich, 2016). In xenobiology, UAAs are broadly categorized into two classes: (i) UAAs with structures similar to those of natural amino acids, often referred to as analogs or derivatives (Agostini et al., 2017) and (ii) UAAs with structures significantly different from those of natural amino acids, known as surrogates (Lepthien et al., 2008). Two main approaches are employed to synthesize UAAs: chemical synthetic routes and biocatalytic routes.

Chemical synthesis offers the advantages of synthesizing UAAs with diverse structures and wide applicability. Chemical synthetic routes are employed to obtain not only individual UAA structures but also dipeptide structures. The propeptide strategy stands out as an effective method to enhance the uptake efficiency of UAAs. The chemical synthesis of dipeptide structures facilitates the transport of poorly bioavailable UAAs into cells through ATP-binding box transporters (Fickel and Gilvarg, 1973; Parrish et al., 2012). Luo et al. prepared two dipeptides, H_2_N-lysine-phosphotyrosine-COOH (Lys-pTyr) and H_2_N-lysine-4-phosphomethyl-l-phenylalanine-COOH (Lys-Pmp), which led to an increase in the cytoplasmic concentration of these two UAAs in *E*. *coli* (Luo et al., 2017a). However, chemical synthesis lacks enantioselectivity and stereoselectivity, involves tedious steps and results in moderate yields (Narancic et al., 2019). In contrast, enzymatic asymmetric synthesis of chiral amino acids has the benefits of simple reaction steps and high enantioselectivity, conversion, and yield (Xue et al., 2018). Protein engineering has enabled the application of enzymes in UAA synthesis, overcoming their low stability and limited substrate range found in nature. Large-scale biosynthesis of UAAs is currently limited by its dependence on cofactors, but this challenge could be addressed by improving cofactor recycling methods (Narancic et al., 2019).

#### Blank codons and orthogonal aaRS/tRNA pair

**Figure 2.**
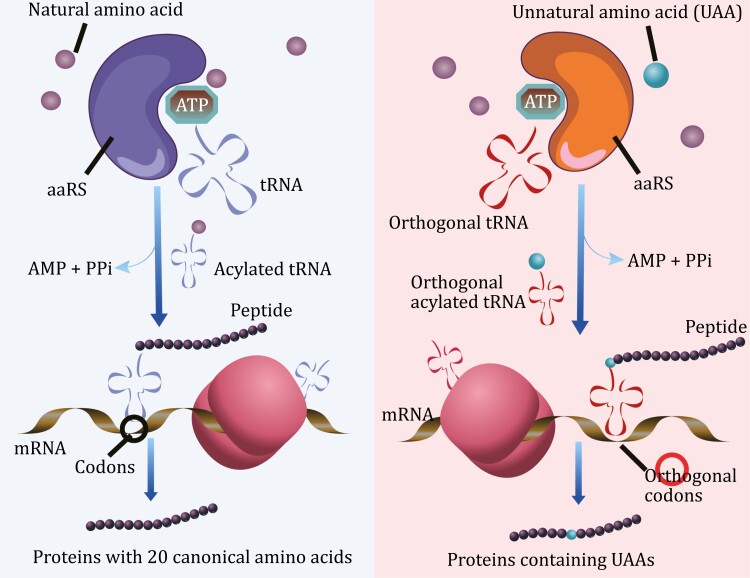
Overview of the working process and key components of the suppression system. Orthogonal tRNA synthetases (aaRSs) are responsible for aminoacylating their corresponding orthogonal tRNAs through UAAs. The aminoacylated tRNA then recognizes the blank codon on the ribosome, leading to the site-specific incorporation of UAAs into target proteins.

Using auxotrophic strains to reduce the concentration of natural amino acids can help prevent the incorporation of nonspecific amino acids, even if they are not targeted to a specific site in the protein of interest (POI). However, even in auxotrophic strains, amino acids may still be present in the cellular pool and can be preferentially incorporated by aminoacyl-tRNA synthetase (aaRS)/tRNA pairs, leading to a lower efficiency of UAA incorporation. To achieve site-specific incorporation of UAAs into target proteins, a strategy using nonsense suppression is needed. This involves mutating the target site to a suppressor codon and introducing orthogonal aaRS/tRNA pairs into cells (Fig. 2).

The most widely used suppressor codon today is the amber nonsense codon (TAG), which has proven to be an effective tool for studying protein structure and manipulating protein function (Wang et al., 2001; Chemla et al., 2018). Additionally, the emergence of quadruplet codons in recent years has provided new ideas for incorporating UAAs (Neumann et al., 2010; Wang et al., 2012; Hankore et al., 2019). Regarding orthogonal aaRS/tRNA pairs, this means that each specific aaRS is specific only to its homologous amino acid and only recognizes its cognate isoacceptor tRNA (iso-tRNA) set, while the iso-tRNA set is only recognized by the given aaRS (Chin, 2006). The orthogonality of the suppression system in GCE ensures that site-specific incorporation of UAAs cannot be disturbed by the host’s endogenous aaRS/tRNA pair or other natural amino acids, greatly improving the accuracy and reliability of UAA incorporation into proteins (Mukai et al., 2017).

In eukaryotic systems, the common orthogonal aaRS/tRNA pairs include PylRS/tRNA^Pyl^, *Ec*TyrRS/tRNA^Tyr^, *Ec*LeuRS/tRNA^Leu^, and *Ec*TrpRS/tRNA^Trp^. The typical UAAs recognized by each orthogonal pair are listed in Table 1. PylRS/tRNA^Pyl^ and *Ec*TyrRS/tRNA^Tyr^ are the two most frequently used aaRS/tRNA pairs in eukaryotic systems. Owing to its high substrate side chain promiscuity and high tolerance for mutations in the substrate binding pocket (Wan et al., 2014), PylRS, derived from archaea of the family *Methanosarcinaceae*, possesses flexible active sites and has been widely shown to be orthogonal in bacteria and eukaryotes (Tharp et al., 2018). Therefore, PylRS is one of the most frequently applied aaRSs in eukaryotes. The *Ec*TyrRS/tRNA^Tyr^ pair is also an aaRS pair with good orthogonality and was first developed by the Schultz group in 2003 (Chin et al., 2003). TyrRS has a spacious active site and can be mutagenized relatively easily to favor the incorporation of bulky UAAs (Crnković et al., 2019). *Ec*LeuRS/tRNA^Leu^ and *Ec*TrpRS/tRNA^Trp^ were developed in 2004 and 2017, respectively, for the incorporation of derivatives of leucine, methionine and cysteine (Wu et al., 2004) or tryptophan derivatives (Italia et al., 2017a). It is worth mentioning that in a recent study, Osggood *et al*. reported that the *Ec*TrpRS/tRNA^Trp^ pair, unlike the other three common aaRS/tRNA pairs, exhibited a higher suppression efficiency for the TGA codon than for the TAG codon in mammalian cells. This property makes it an excellent tool for establishing a dual-UAA incorporation system (Osgood et al., 2023).

**Table 1.** Four common orthogonal aaRS/tRNA pairs and the typical UAAs in eukaryotes.

Orthogonal system	Origins	Codons	Typical UAAs	References
PylRS/tRNA^Pyl^ pair	*Mj.* *Mm.*	UAG/GCA/AGC/CUG/AUG/ UAA/GUG/AAG/GAG/UAC/ UGG/AGGA/CUAG/UAGA	Lysine derivatives Phenylalanine derivatives	Mukai et al. (2008), Chatterjee et al. (2013b), Niu et al. (2013), Xiao et al. (2013), Elliott et al. (2014), Mills et al. (2021), and Xi et al. (2022)
TyrRS/tRNA^Tyr^ pair	*E*. *coli*	UAG	Tyrosine derivatives Phenylalanine derivatives	Chen et al. (2007), Parrish et al. (2012), Palzer et al. (2013), Shao et al. (2015), and Mattheisen et al. (2023)
LeuRS/tRNA^Leu^ pair	*E*. *coli*	UAG	Methionine derivatives Cysteine derivatives Lysine derivatives Alanine derivative	Wu et al. (2004), Takimoto et al. (2010), Wu *et al*. (2022b), and Mattheisen et al. (2023)
TrpRS/tRNA^Trp^ pair	*E*. *coli, Bs.*	UAG/UGA	Tryptophan derivatives	Zhang et al. (2004), Italia et al. (2017a), and Osgood et al. (2023)

#### Challenges and optimization

##### Biosynthesis of UAAs

One of the key challenges in biosynthesizing UAAs is the difficulty of the heterologous expression of certain enzymes required for enzymatic catalysis in surrogate hosts. However, the cell-free protein system (CFPS) offers a new solution for the biosynthesis of UAAs (Tian et al., 2022). In a recent study, five enzymes in a biosynthetic pathway were successfully expressed using a CFPS, leading to the catalytic conversion of the lysine substrate to various lysine derivatives (Chen et al., 2023).

##### Utilization of UAAs

Numerous studies have reported methods to enhance the transmembrane efficiency of UAAs, such as utilizing a propeptide strategy in which the target UAA and its nonhydrolyzable analogs are incorporated into a dipeptide. This dipeptide is transported into cells and then hydrolyzed by nonspecific intracellular peptidases, thereby increasing the transmembrane efficiency of UAAs (Luo et al., 2017a). Another approach involves engineering bacterial organisms with an improved UAA uptake capacity by screening periplasmic binding protein mutants for UAA recognition (Ko et al., 2019).

When incorporating UAAs into eukaryotic cells or multicellular organisms, it is a common practice to administer high concentrations of exogenous UAAs to compensate for their poor integration efficiency in these systems. Autonomous biosynthesis systems for UAAs were initially developed for bacterial cells (Mehl et al., 2003; Chen et al., 2018). More recently, similar systems have been reported to replace the need for high concentrations of exogenous UAAs in eukaryotic cells and zebrafish (Wu et al., 2022a). Tyrosine sulfation, with the formation of sulfotyrosine (sTyr), is a PTM of proteins with low membrane permeability. To address the issue of low membrane permeability, the Xiao group utilized bioinformatics and computational methods to identify a sulfotransferase for the biosynthesis of sTyr. After further optimization of this sulfotransferase, cells capable of autonomous production of sTyr were constructed, which ameliorated the problem of inefficient incorporation caused by the low membrane permeability of sTyr (Chen et al., 2022a). This kind of autonomous biosynthetic UAA incorporation may facilitate the application of GCE in multicellular organisms. While there are various methods to enhance the utilization of UAAs, there is currently no universal and convenient approach to improve UAA uptake efficiency in eukaryotes.

##### Directed evolution of aaRSs and tRNAs

To achieve orthogonality, aaRS evolution is required to remove the affinity of aaRSs for cognate amino acids and to improve the acylation efficiency of UAAs. The engineering of aaRSs includes complex analysis and redesign of the aaRS active sites, as well as mutagenesis of more than 15 residues around the active sites to evolve aaRSs with better UAA specificity (Crnković et al., 2019). Most evolved aaRS mutants contain only a few mutations that can enhance their activity against specific UAAs. Therefore, it is feasible to generate aaRSs capable of aminoacylating specific UAAs by building a library of aaRS mutants and identifying small improvements in aaRSs after high-throughput screening (Liu and Schultz, 2010; Crnković et al., 2019). The evolution of aaRSs using reporter assays, in which successful incorporation of a UAA into proteins serves as a marker of successful UAA aminoacylation, has been widely used (Liu et al., 1997; Santoro and Schultz, 2003). In addition, a two-step process involving positive and negative rounds to further evaluate and improve the orthogonality of an aaRS/tRNA pair toward a UAA has been extensively used (Santoro et al., 2002; Melançon and Schultz, 2009). The phage-assisted continuous evolution (PACE) technique has also been applied to the directed evolution and screening of aaRSs, enabling the rapid identification of PylRS mutants with improved kinetic parameters (Esvelt et al., 2011).

Functional expression of orthogonal tRNAs in eukaryotic systems is challenging owing to differences in tRNA transcription between prokaryotes and eukaryotes. Orthogonal tRNAs are typically derived from prokaryotes, wherein tRNA transcription is driven by upstream promoters. In contrast, in eukaryotes, transcription is initiated by promoter elements, known as A- and B-boxes within a tRNA gene, which are absent in prokaryotes. Therefore, it is difficult to express prokaryotic-derived orthogonal tRNAs in eukaryotes (Wang, 2017). In 2007, the Wang group reported that type-3 Pol III mammalian promoters (such as H1, 7SK, U6 snRNA, etc.) in combination with 3ʹ-flanking sequences could drive prokaryotic tRNA expression in mammalian cells (Wang et al., 2007). They also identified two Pol III yeast promoters for the expression of prokaryotic tRNAs in yeasts (Wang and Wang, 2008, 2012). Additionally, the expression level of a tRNA can be increased by repeating these tRNA expression cassettes (Coin et al., 2011).

### *In vitro* eukaryotic platform

#### Yeast

The successful incorporation of UAAs in *E*. *coli* has led to further exploration of the applications of the GCE strategy in eukaryotic cells for the manipulation or study of eukaryotic cellular processes. In 2003, Chin and his colleagues overcame the obstacles posed by the translation machinery used in *E*. *coli*, which could not be applied to eukaryotic expression systems. They first incorporated five UAAs in *S. cerevisiae* (Chin et al., 2003). However, the low efficiency of UAA incorporation in yeast limits the effective application of this technology to the eukaryotic expression platform. The nonsense-mediated mRNA decay (NMD) pathway in yeast always leads to the rapid degradation of an mRNA that contains premature stop codons (Mitchell and Tollervey, 2003). The incorporation of UAAs into proteins can be affected when NMD occurs, leading to a shorter lifetime of the target mRNA and a lower protein yield. To address this problem, Wang et al. (2008) produced an NMD-deficient yeast strain and obtained a high yield compared to that in wild-type yeast in 2008. In addition, they optimized the tRNAs by applying internal leader Pol III yeast promoters to improve the efficiency of UAA incorporation.

The generation of the synthetic yeast *S. cerevisiae* genome 2.0 (Sc2.0) has provided a more diverse and promising eukaryotic expression platform for GCE. Sc2.0 enables the rapid generation of genetic variants with a large number of genomic rearrangements and modifications by using the Synthetic Chromosome Rearrangement and Modification by LoxPsym-mediated Evolution (SCRaMbLE) system mediated by loxP sites (Wu et al., 2018a). This facilitates rapid optimization of the genomic background, thereby improving the efficiency of UAA integration. Sc2.0 also provided a useful model for exploring the limitations of translation in eukaryotes (Sanders et al., 2022).

#### Mammalian cell lines

Mammalian cellular models are widely used research objects in the study of physiological and pathological processes. GCE is a powerful tool that can be applied in mammalian cells to dynamically monitor these processes, offering valuable insights for researchers.

The incorporation of UAAs into mammalian cells faces two major obstacles. The first is the low expression efficiency of prokaryotic-derived tRNAs in mammalian cells (Wang, 2017), while the second is the limited availability of engineered aaRS/tRNA pairs screened by the selection system, which restricts their incorporation into cells (Italia et al., 2017b). To overcome these difficulties, researchers have employed several strategies, including optimizing the expression of tRNAs by introducing the type-3 polymerase III promoter (Wang et al., 2007) and using transfer strategies (Zheng et al., 2017), increasing the affinity of aaRS/tRNA pairs (Takimoto et al., 2009), and constructing a special selection system for aaRS/tRNA pairs known as ATM (Italia et al., 2017a). Stem cells are characterized by their ability to differentiate into distinct cell lineages through a complex signaling network that regulates self-renewal and pluripotency. This feature has made stem cells a valuable tool for developing several therapeutic strategies (Hoang et al., 2022). However, in the case of terminally differentiated, nondividing cells, such as neurons, introducing UAAs into proteins can modulate their protein function and monitor their physiological or pathological cellular processes. Obtaining such mature cells through conventional methods, which involve isolation from living tissues, can be challenging as it is difficult to stably introduce exogenous material into them (Shen et al., 2011). To address this, researchers have explored incorporating UAAs into proteins in stem cells and inducing their differentiation into mature cells for research purposes.

This idea raises new questions as achieving stable expression in stem cells is challenging, and commonly used transient transfection methods are not applicable. Transient transfection of stem cells is known to be inefficient (Wang et al., 2007), and the differentiation process of stem cells is often lengthy, leading to a loss of the orthogonal aaRS/tRNA pair if transient transfection is used. This creates uncertainty regarding the successful incorporation of UAAs into terminally differentiated nondividing cells (Shen et al., 2011). Therefore, stable expression of exogenous aaRS/tRNA pairs in stem cell chromosomes is necessary to study the entire differentiation process of stem cells using UAAs. Shen and colleagues (2011) utilized a lentiviral vector to stably incorporate fluorescent UAAs into the voltage-dependent membrane lipid phosphatase of the neural stem cell line HCN-A94 and induced stem cell differentiation. The study also showed that UAA incorporation did not affect the differentiation process of stem cells. Furthermore, the application of GCE in this study helped reveal conformational changes in voltage-sensitive domains during depolarization of the cell membrane.

### *In vivo* eukaryotic platforms

#### Invertebrate animals

##### Caenorhabditis elegans

*Caenorhabditis elegans* has been an important model system for understanding mutant phenotypes at the cellular level since the 1970s because of its sequenced genome and mapped lineage for each cell from embryogenesis through postembryonic development (Sulston and Horvitz, 1977; Kimble and Hirsh, 1979). With its excellent optical transparency (Wang and Audhya, 2014; Kinser and Pincus, 2017), *C*. *elegans* has been extensively utilized in studies of photocaging (Alexpandi et al., 2019; Prasanth et al., 2020), photocrosslinking (Burnett et al., 2018), and fluorescent UAAs (Chang et al., 2013). In 1985, researchers demonstrated that *C*. *elegans* tolerated amber stop codon suppression (Hodgkin, 1985). Furthermore, *C*. *elegans* is small and easy to culture, making it an ideal candidate for GCE. Until recently, *C*. *elegans* was used as the primary multicellular biological platform for GCE.

To incorporate UAAs into *C*. *elegans*, researchers used a type 3 Pol III promoter (Chang et al., 2013) or the the RNase P RNA (*rpr-1*) promoter (Davis et al., 2021) to express orthogonal tRNAs from *E*. *coli*. Previous studies have shown that orthogonal aaRS/tRNA pairs and reporters were transiently expressed on extrachromosomal arrays in *C*. *elegans,* and the expression was maintained through the use of antibiotics (Frøkjaer-Jensen et al., 2008). However, this approach leads to a UAA-independent amber codon read-through and a low success rate in constructing mutant *C*. *elegans* (Wang, 2017). Genomic integration has significantly increased the success rate of creating transgenic *C*. *elegans* that exhibits a high degree of UAA dependence (Parrish et al., 2012). This phenomenon highlights that GCE in *C*. *elegans* requires the stabilization of orthogonal aaRS/tRNA pairs in the genome, ensuring genetic uniformity and inheritance.

A study conducted by Parrish et al. (2012) demonstrated the incorporation of various UAAs in response to the amber stop codon into several proteins in *C*. *elegans* by directly evolving the *E*. *coli*-derived tyrosyl and amber leucyl suppressor aaRS/tRNA pairs. The NMD pathway is a major factor that impedes efficient UAA incorporation in *C*. *elegans*. To overcome this issue, researchers disrupted the NMD pathway by knocking down *smg-1*, which resulted in efficient incorporation of UAAs in *C*. *elegans* (Parrish et al., 2012). Another study aimed to eliminate the interference from the NMD pathway by crossing NMD-deficient smg-2(e2008) worms with worms expressing a reporter, which led to an increased GFP reporter expression signal (Greiss and Chin, 2011).

##### Drosophila melanogaster

Similar to *C*. *elegans*, *D. melanogaster*, as the most established metazoan model organism, has a sequenced genome (Adams et al., 2000) and was shown to be tolerant of amber stop codon suppression in a previous study (Laski et al., 1989). Additionally, it has advantages as a potential platform for expanding the genetic code, including a short generation time, an established tool for creating transgenic flies, and control of transgene expression (Laski et al., 1989; Garza et al., 1990; Washburn and O’Tousa, 1992). Consequently, in 2012, Bianco et al. (2012) investigated the incorporation of UAAs into *D*. *melanogaster* by expressing PylT from a type 3 Pol III promoter and using an orthogonal PylRS/tRNA system to successfully integrate two UAAs into *D*. *melanogaster*.

#### Vertebrate animals

##### Zebrafish

Zebrafish is a widely used model organism in the study of vertebrate development and disease owing to its high fecundity and short generation time. Its excellent optical transparency during embryonic stages enables noninvasive *in vivo* imaging with high spatial and temporal resolutions, making zebrafish a powerful tool for investigating gene regulation and networks. In summary, zebrafish is an outstanding noninvasive optical model for biological research.

To expand the genetic code in zebrafish, the PylRS system was introduced, and photocaged UAAs were incorporated into zebrafish embryos to optically control mitogen-activated proteinkinase kinase 1 (MEK1) activation, affecting the MEK/extracellular signal-regulated kinase (ERK) pathway and observing the role of the activation of the MEK/ERK pathway at different stages of zebrafish development (Liu et al., 2017). Subsequently, some investigators successfully incorporated several UAAs that had never been used in the zebrafish expression system (Syed et al., 2019). It has been suggested that the application of the zebrafish expression system as a relatively new platform for GCE has gradually matured.

##### Mouse

*Mus musculus* is a commonly used laboratory mouse that is widely employed as a mammalian model in the field of life sciences for physiological, pathological, and pharmacological studies (Chen et al., 2017). In 2013, the GCE technique was first applied to the embryonic mouse neocortex *in vivo* to genetically incorporate a photoreactive UAA into Kir2.1, an inwardly rectifying potassium channel, thereby regulating K^+^ channel switching to control neuronal firing (Kang et al., 2013). A major challenge for UAA incorporation in mice, as in other *in vivo* platforms, is to find an efficient way to deliver and express the necessary components for UAA incorporation. In the aforementioned work, researchers employed uterine electroporation to deliver the *E*. *coli*-derived LeuRS/tRNA_CUA_ pair into the mouse neocortex.

However, there is still a need to develop widely applicable and efficient gene delivery methods for *in vivo* platforms. In 2016, Ernst et al. (2016) reported the use of adeno-associated viral (AAV) vectors to deliver the components of the suppression system to live mouse brains. This delivery method offers the advantage of site-specific and efficient incorporation of different UAAs through the injection of AAV vectors. However, stable gene expression cannot be achieved with this approach, and the expression of aaRS/tRNA pairs is transient. One year later, a transgenic mouse capable of stably expressing AcKRS/tRNA pairs was developed to temporally and spatially control protein acetylation in different mouse organs (Han et al., 2017). In this study, two transgenic mice that expressed PylRS/tRNA and GFP39TAG/tRNA were developed. Furthermore, they were crossed to generate double-heterozygous animals carrying the components of the suppression system and the reporter gene GFPuv. When these double-heterozygous mice were crossed with a transgenic mouse carrying an amber stop codon, a UAA was successfully incorporated into multiple organs through daily intraperitoneal injection alone (Han et al., 2017). This strategy provides a convenient system for the expansion of the genetic code *in vivo*, but establishing a stable mouse line is time consuming.

#### Plants

Using plants instead of microbial fermentation or animal cell cultures to produce therapeutic proteins has been proposed as an attractive approach. Expanding the genetic code in plants could lead to safer, more productive, and less costly recombinant therapeutic processes. Arabidopsis, a model organism in plant biology, was the first plant species to have its genome sequenced. In 2013, Li et al. attempted to introduce UAAs into *Arabidopsis thaliana*. To circumvent the NMD pathway, researchers selected a mutant lacking the UPF1 gene for their study, as UPF1 is a key regulator of the NMD pathway in *A. thaliana* (Li et al., 2013). Furthermore, the authors chose the type 3 Pol III Arabidopsis promoter 7SL4 to express tRNA_CUA_^Pyl^. In this work, the UAA *N*-ε-acryllysine (AcrK) was site-specifically incorporated into a reporter in *A. thaliana* (Li et al., 2013). This approach could be useful for expanding genetic coding in other plants in the future.

## Applications of GCE

### Basic research

#### Protein activity switch

##### Optical control

To manipulate biological processes naturally and precisely, scientists require means to regulate protein activity. Traditional methods of regulating protein activity include using the tetracycline (Tet) system (Kozlova and Berens, 2012), gene recombination systems such as Cre-loxP (Kim et al., 2018), or siRNAs (Stokman et al., 2010; Friedrich and Aigner, 2022). However, optical control of protein function provides noninvasive and precise spatiotemporal regulation compared to these approaches. The incorporation of light-responsive amino acids into proteins is an important way to optically regulate protein functions. Over the past few decades, several studies have demonstrated the effectiveness of optical control of protein function. With recent research advancements, significant breakthroughs have been made in the application of this approach in eukaryotes.

One approach to optically regulate protein function is to incorporate UAAs containing a photolabile protecting group (photocage) at specific sites of proteins. The activation state of proteins can be modulated by light of specific wavelengths, depending on the nature of the photocage group. The *O*-nitrobenzyl (ONB) group is a common photocage group (Wu et al., 2004), and the ONB-caged versions of several UAAs are stable under physiological conditions. However, they require high-intensity UV light (250–365 nm) to remove the cage group, which can cause cellular damage. To overcome this limitation, modifications to the structure of cage groups have been made. For instance, dimethoxy-nitrobenzyl (DMNB)-caged UAAs have high quantum yields and can be deprotected in living cells by visible blue light (Lemke et al., 2007). UAA modified with these photocage groups has been incorporated into mammalian cells, *C*. *elegans* and zebrafish to precisely regulate the function of various proteins, including proteases, nucleases, kinases, and the Cas9 protein. Table 2 summarizes the applications of these photocaged UAAs.

**Table 2.** List of photocaged amino acids incorporated in eukaryotes.

Amino acid	Structure	Eukaryotes	aaRS/tRNA pair	Application	References
Caged lysine	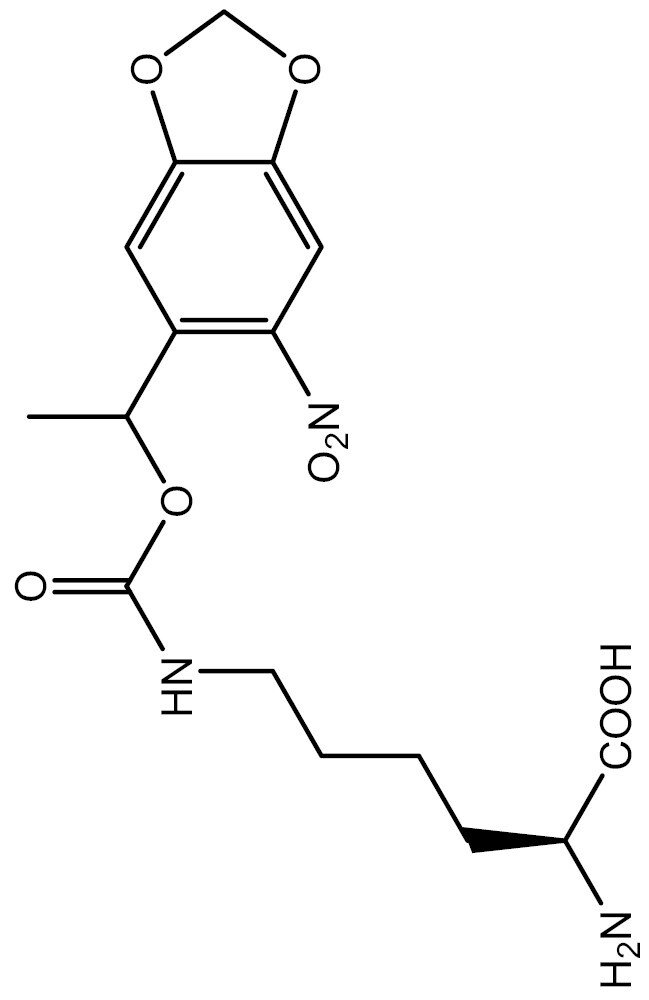	HEK293T	PylRS/tRNA^Pyl^	Cre recombinase	Luo et al. (2016a, b)
				T7 RNA polymerase	Hemphill et al. (2013)
				MEK1	Gautier et al. (2011)
				Isocitrate dehydrogenase	Walker et al. (2016)
				Nuclear localization signal (NLS) of the transcription factor SATB1	Engelke et al. (2014)
				NLS of nucleoplasmin and the tumor suppressor p53	Gautier et al. (2010)
				CRISPR associated (Cas) proteins	Hemphill et al. (2015)
		HEK-TCR cell (Created by HEK293T and stably express 1G4 TCRαβ chains and CD3γδɛζ chains)		A tyrosine kinase LCK	Liaunardy-Jopeace et al. (2017)
		*C. elegans*		Cre recombinase	Davis et al. (2021)
				Cre recombinase	Xi et al. (2022)
	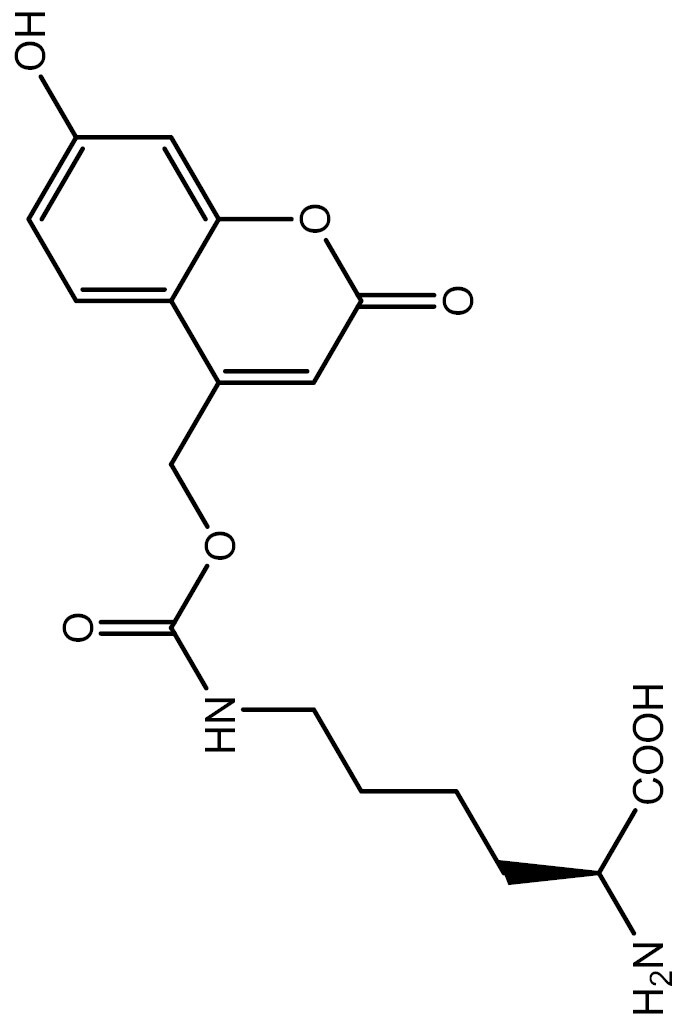	HEK293T		EGFP and Fluc	Luo et al. (2014)
				SH2 domain-containing protein tyrosine phosphatase-2	Ryan et al. (2022)
				MAPK phosphatase	Courtney and Deiters (2019)
		Zebrafish embryos		MEK1	Liu et al. (2017)
				Cre recombinase	Brown and Deiters (2019)
	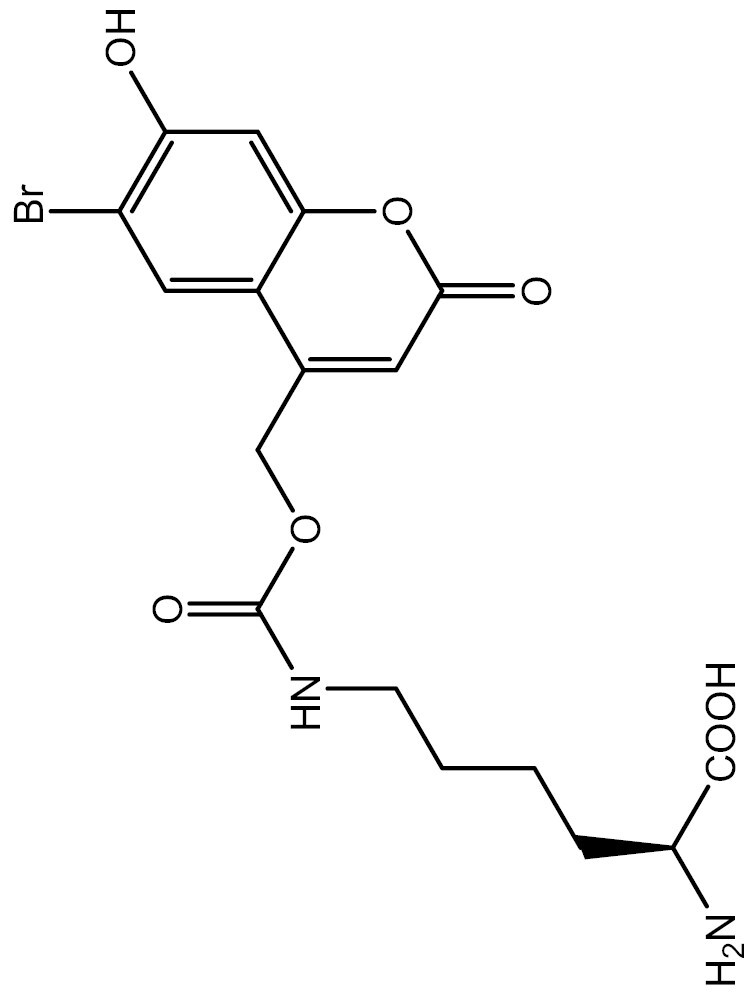	HEK293T		EGFP and Fluc	Luo et al. (2014)
Caged tyrosine	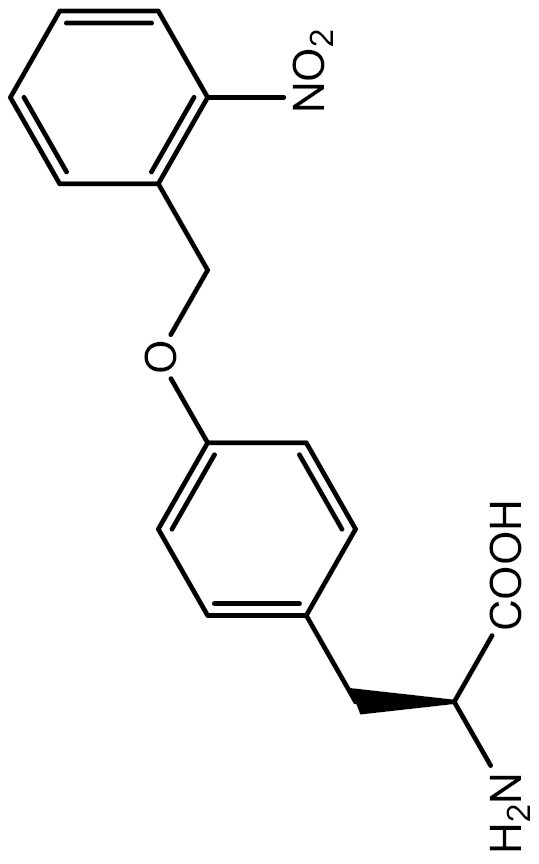	HEK293T	PylRS/tRNA^Pyl^	EGFP and Fluc	Luo et al. (2017b)
				Cre recombinase	Edwards et al. (2009)
				T7 RNA polymerase	Chou et al. (2010)
				Zinc-Finger nucleases	Chou and Deiters (2011)
				STAT1 phosphorylation	Arbely et al. (2012)
				Toll-like receptor 8	Yang et al. (2022)
		HEK293T, PC12 cell line and SH-SY5Y cell		Tyrosine kinase A	Zhao et al. (2020)
	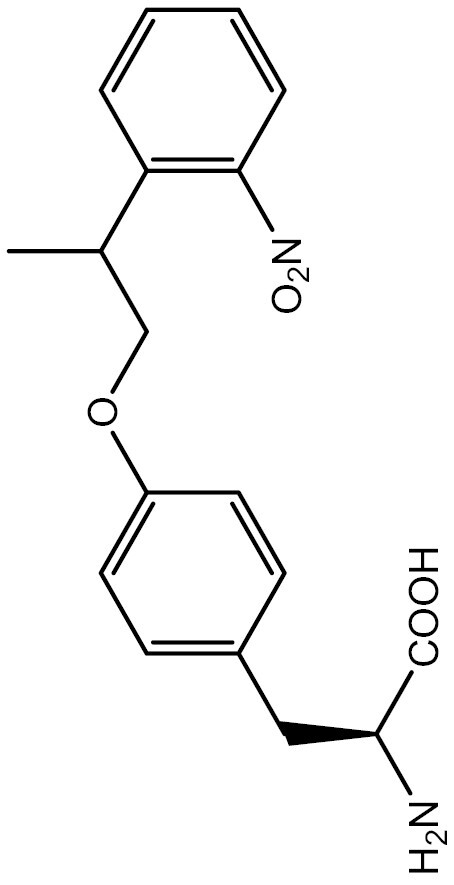	HEK293T		EGFP and Fluc	Luo et al. (2017b)
	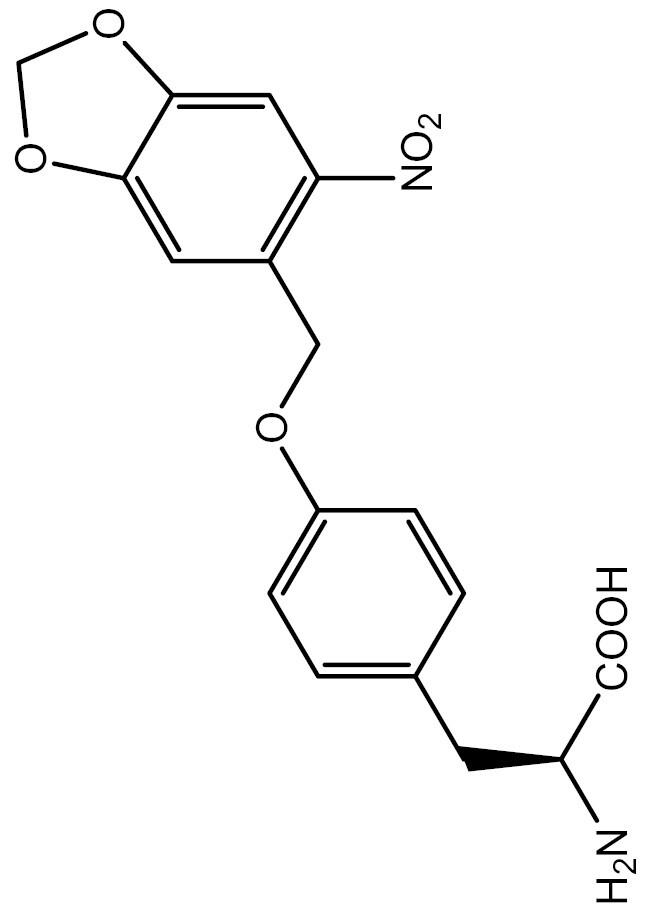	HEK293T		EGFP and Fluc	Luo et al. (2017b)
				SH2 domain-containing protein tyrosine phosphatase-2	Ryan et al. (2022)
				Toll-like receptor 8	Yang et al. (2022)
	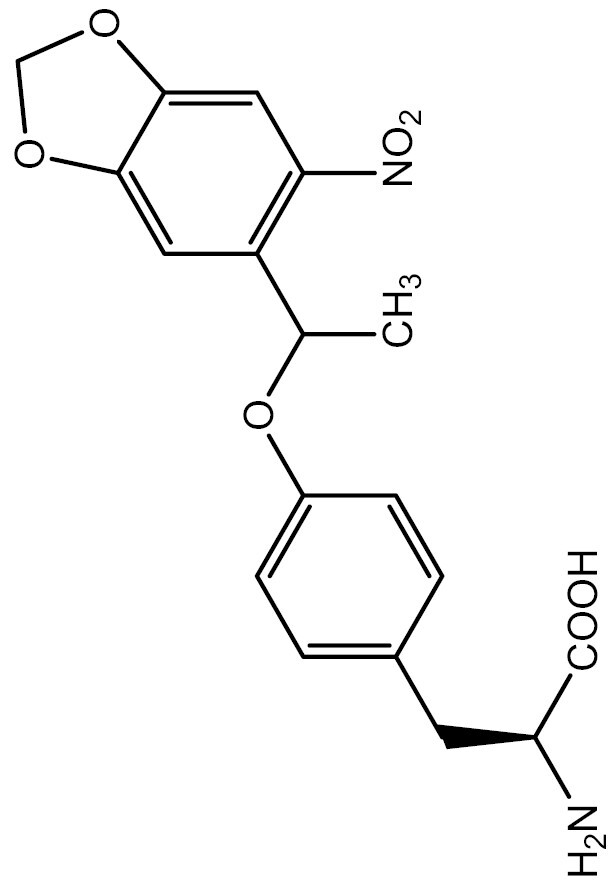	HEK293T		EGFP and Fluc	Luo et al. (2017b)
				Cre recombinase	Luo et al. (2016a)
Caged cysteine/ selenocysteine/ homocysteine	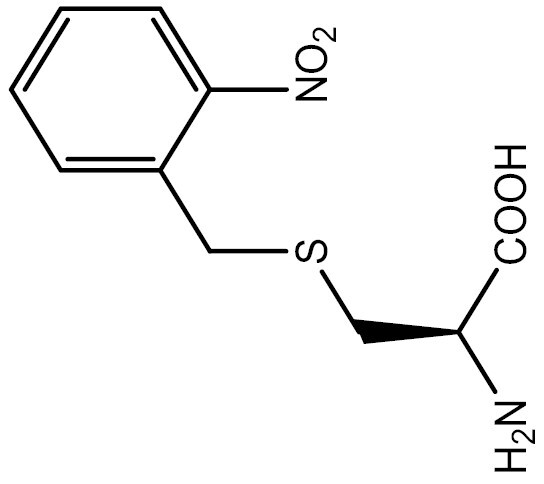	HEK293T	PylRS/tRNA^Pyl^	TEV protease	Nguyen et al. (2014)
	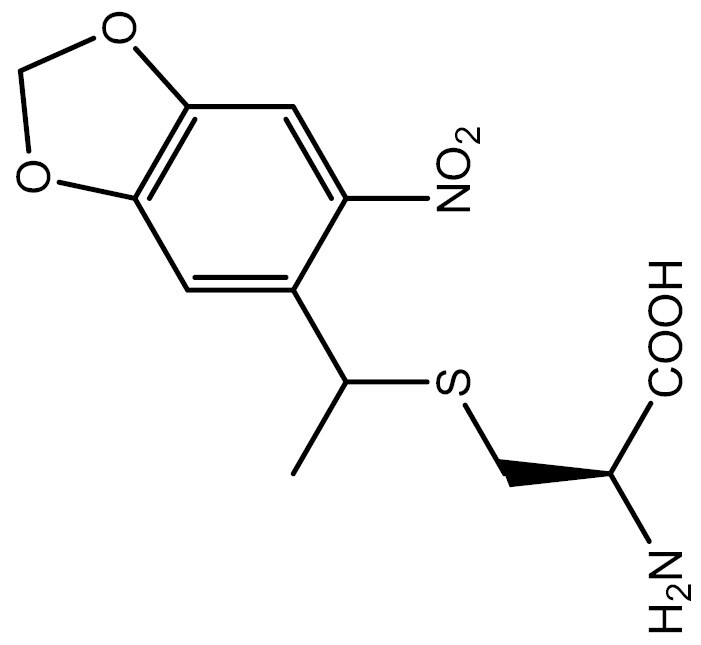	HEK293T	PylRS/tRNA^Pyl^	TEV protease	Nguyen et al. (2014)
		*C. elegans*	PylRS/tRNA^Pyl^	Casepase-3	Xi et al. (2022)
	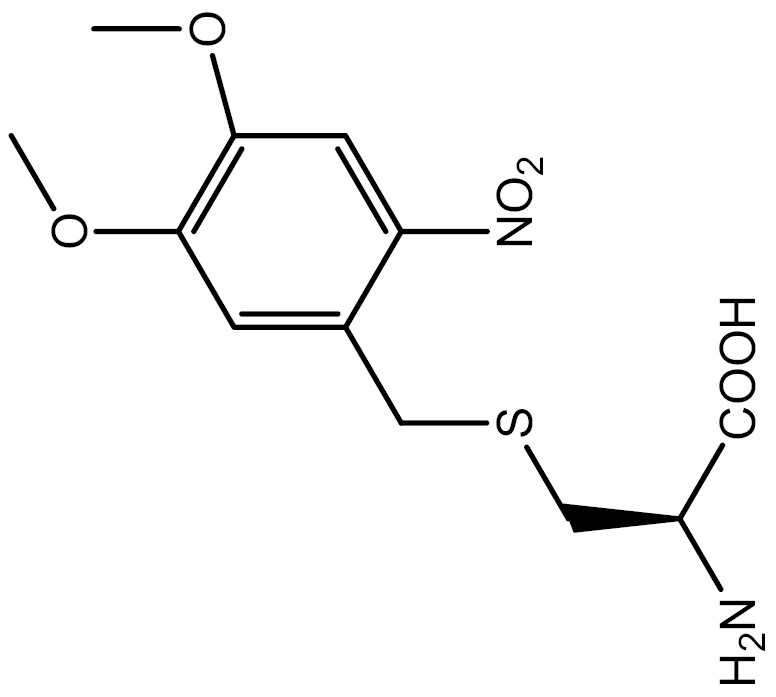	HEK293T	LeuRS/tRNA^Leu^	MAPK phosphatase	Courtney and Deiters (2019)
				Src	Ren et al. (2015a)
		Mouse		Kir2.1	Kang et al. (2013)
	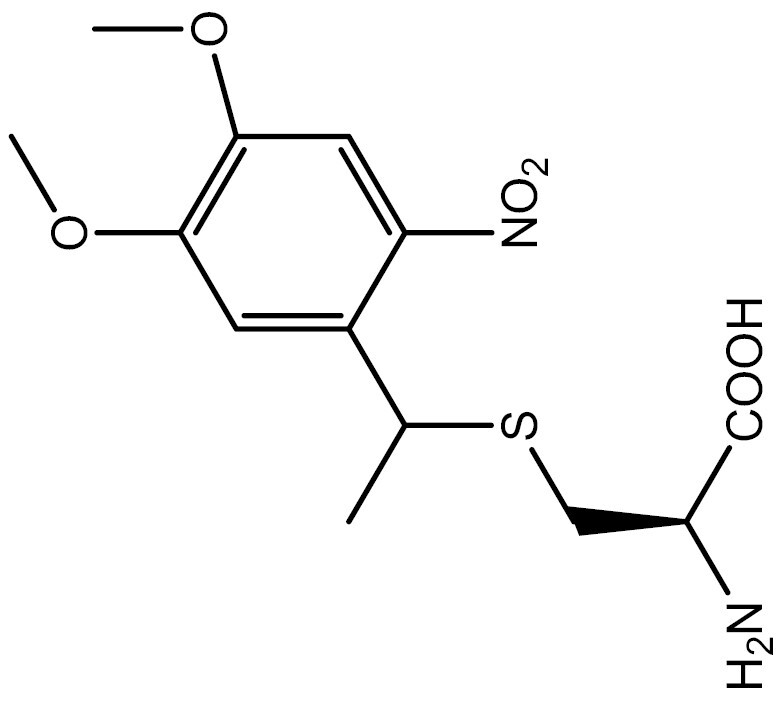	HEK293T	LeuRS/tRNA^Leu^	Src	Ren et al. (2015a)
	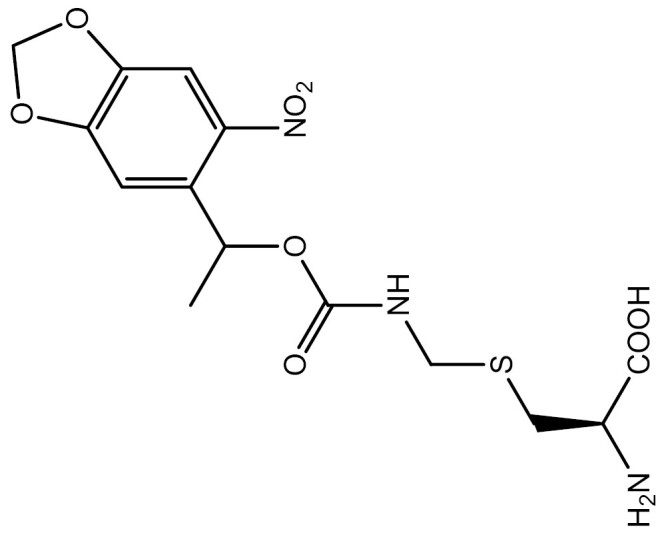	HEK293T	PylRS/tRNA^Pyl^	EGFP and Renilla luciferase	Uprety et al. (2014)
	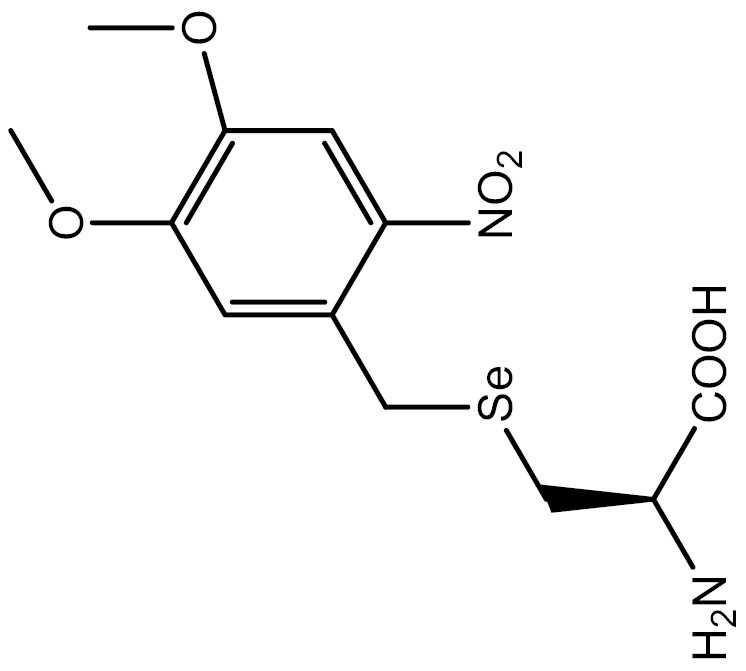	HEK293T	LeuRS/tRNA^Leu^	EGFP	Peeler et al. (2020)
	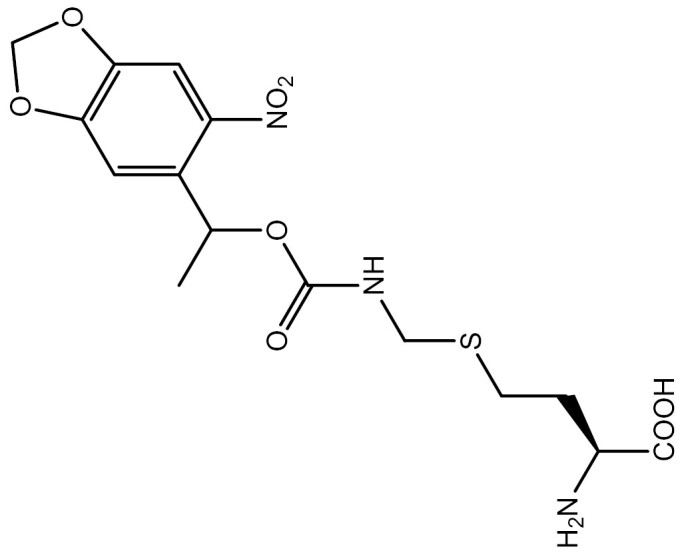	HEK293T	PylRS/tRNA^Pyl^	EGFP and Renilla luciferase	Uprety et al. (2014)
Caged histidine	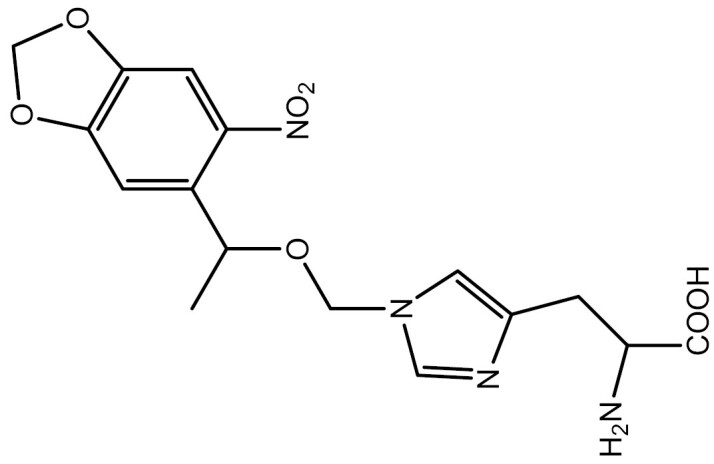	Zebrafish embryos	PylRS/tRNA^Pyl^	Cre recombinase and the single-chain variant viral protease from the hepatitis C virus	Brown et al. (2023a)

Although the incorporation of photocaged amino acids has shown advantages in rapidly and precisely regulating protein function, their irreversible activation based on the decaging reaction limits their application in studying protein activation-deactivation cycles under physiological conditions (Courtney and Deiters, 2018). Moreover, targeting a single residue to modulate protein function has limited effects, as protein function often involves the structure of protein domains and conformation (Beharry and Woolley, 2011). To overcome these limitations, photoswitchable amino acids (PSAAs) have been developed. PSAAs containing an azobenzene moiety can covalently bind to a cysteine residue in the protein domain to form a photocontrollable bridge *in situ*, allowing light-inducible control of protein conformation (Hoppmann et al., 2015; Hoppmann and Wang, 2019). Furthermore, the azobenzene moiety in a PSAA can reversibly change its conformation under two wavelengths of light, making the regulation of protein activity reversible (Aemissegger and Hilvert, 2007). In 2006, a photoswitchable azobenzene amino acid was successfully encoded in *E*. *coli* (Bose et al., 2006). Earlier studies incorporated amino acids with azobenzene moieties into proteins such as catabolite activator protein (Bose et al., 2006) and horseradish peroxidase (Muranaka et al., 2002), which played well-controlled roles. This strategy has also been applied to eukaryotic systems. The Chin group used this strategy for their research and reported the rapid and selective regulation of specific MEK isoenzymes in mammalian cells (Tsai et al., 2015). The Wang group subsequently achieved reversible photoswitching control of glutamate receptors in mammalian cells, thereby proving the feasibility of reversible optical control of neuronal receptors (Klippenstein et al., 2017). Nonetheless, there are several limitations to the widespread application of PSAAs. One limitation is the difficulty in determining suitable PSAA incorporation sites, which requires extensive screening. Current technology cannot guarantee that such sites can be found in all target proteins (Nödling et al., 2019).

##### Small-molecule regulation

**Figure 3.**
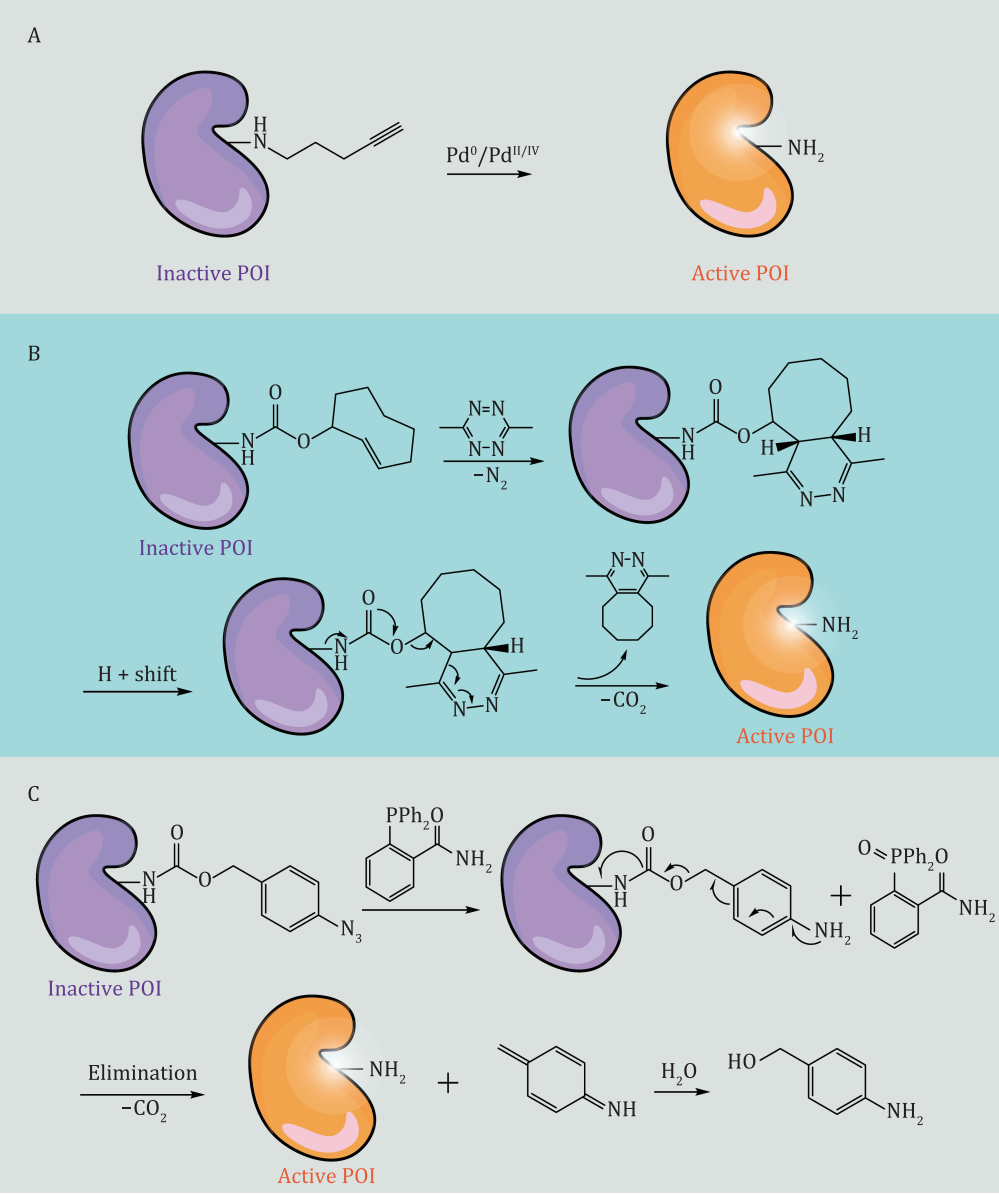
Three primary reactions for removing protective groups from UAAs through small molecule modulation. (A) Utilizing a palladium catalyst to decage propargyloxycarbonyl-caged lysine or allene-caged tyrosine derivatives. (B) Employing the Diels–Alder (invDA) cycloaddition reaction to eliminate the *trans*-cyclooctenyloxycarbonyl (TCO) group. (C) Applying the Staudinger reaction to decage the azidobenzyloxycarbonyl (ABZ) group.

In addition to incorporating light-responsive UAAs, protein function can also be controlled by introducing small molecule-cleavable caging groups. Currently, there are three main approaches for removing protecting groups from UAAs using small-molecule modulation in eukaryotic systems. The first method involves using palladium catalysts to deprotect propargyloxycarbonyl-caged lysine or allene-caged tyrosine derivatives (Li et al., 2014b; Wang et al., 2016a) (Fig. 3A). While this approach is highly biocompatible, its slow kinetics and low decaging yields limit its generalizability.

The *trans*-cyclooctenyloxycarbonyl (TCO) group is cleaved by tetrazine via Diels–Alder (invDA) cycloaddition, leading to the release of lysine (Li et al., 2014a) (Fig. 3B). Li et al. were the first to investigate this invDA reaction between the TCO-caged Fomc-lysine analog (Fmoc-TCOK) and various tetrazine derivatives. They identified the optimal caging–decaging pair as TCOK-α and Me2Tet, which they used to successfully incorporate and activate the GPF protein in 293T cells (Li et al., 2014a). This work offers a general approach for rapidly switching protein function through small molecules, which could potentially be extended to the decaging of other natural amino acids. Additionally, the high biocompatibility and deep tissue penetration make this method suitable for use in intact animals. In a subsequent study, researchers applied this UAA-decaging toolkit in mice to manipulate kinase activity *in vivo* (Zhang et al., 2016).

The third strategy involves removing the azidobenzyloxycarbonyl (ABZ) group via the Staudinger reaction, which is induced by phosphine and reduces the azide to an amine, leading to self-immolation and exposure of lysine (Fig. 3C). The Staudinger reduction of aryl azides by phosphines has been demonstrated to be highly biocompatible and nontoxic in living cells or organisms in several studies (Agard et al., 2006; Sletten and Bertozzi, 2011; Fang et al., 2019; Fottner et al., 2019). Based on this reaction, the ABZ-lysine derivative *ortho*-azidobenzyloxycarbonyl lysine (OABK) was first used to control protein function in mammalian cells and regulate multiple cellular processes, including bioluminescence (luciferase), protein translocation (nuclear localization sequence) and DNA recombination (Cre) (Luo et al., 2016b). However, OABK has several limitations that hinder its broad application, the most significant of which is its slow kinetics, which makes it challenging to control protein function on short timescales (Luo et al., 2016b). Consequently, the research team improved the structure of OABK by relocating the azide from the *ortho* position to the *para* position to accelerate the self-immolation reaction of substrates (Alouane et al., 2015), and developed a lysine derivative, PABK, with rapid decaging properties (Wesalo et al., 2020). PABK triggered nuclear translocation 81% faster than OABK and successfully achieved small-molecule control of SUMOylation in mammalian cells (Wesalo et al., 2020).

In a recent study, researchers performed small molecule-triggered UAA-decaging reactions in zebrafish embryos (Brown et al., 2023b). They selected two caging–decaging pairs, *N*^ε^-*p*-azidobenzyloxycarbonyl lysine (PABK)/phosphine 2-(diphenylphoshino) benzamide (2DPBM) and TCOK/methyl tetrazine (MeTz), to investigate their incorporation and decaging efficiencies, as well as their effects on the growth of zebrafish. The study showed that although TCOK/MeTz exhibited a good incorporation efficiency and a faster decaging process, most embryos died at a high MeTz concentration (100 μmol/L) (Brown et al., 2023b). Therefore, the researchers further explored PABK/2DPBM. The results demonstrated that the small molecule-triggered decaging of PABK enabled tunable protein activation in DNA recombination and nuclear translocation events via viral recombinase and viral protease. This system was also used to investigate the molecular mechanism of heart defects caused by a RASopathy mutant of NRAS (Brown et al., 2023b).

Overall, the advantage of small molecule-triggered UAA-decaging strategies over traditional small-molecule protein control methods is their greater universality. This is because small-molecule protein activators are usually identified through high-throughput screening or accidental discovery, which is not universally applicable and cannot be directly transferred to other proteins. In contrast to protein function control based on photocaged UAAs, small-molecule-triggered decaging reactions often do not require special photostimulation equipment. Additionally, UV irradiation of live cells or organisms may cause cytotoxicity and alter intracellular signaling pathways. The application of small molecules in decaging reactions tends to lead to better penetration into deep tissues than that observed upon optical activation, making it more feasible for use in intact animals.

#### Posttranscriptional modifications

PTMs cause covalent attachments of specific chemical groups to side chains of nucleophilic amino acids and can be enzymatic or nonenzymatic (Lee et al., 2023). These modifications greatly enrich the structural and functional diversity of the proteome in cells. PTMs are mediated by enzymes and can affect protein functions, including subcellular localization, stability, activity, and interactions (Barber and Rinehart, 2018). Aberrant PTMs are closely related to many human diseases, and understanding their functional significance in disease can deepen our understanding of diseases and help develop effective therapeutic strategies. Various approaches have been applied to study PTMs, such as replacing serine with glutamate in proteins (Chang et al., 2014), but these methods cannot mimic the stereoscopic effects of PTMs on proteins (Chen and Tsai, 2022). Although enzymes can be used to regulate the PTM process in cells, achieving site-specific modifications can be challenging (Zhang et al., 2016). While chemical synthesis can achieve site-specific modifications, it is difficult, and delivering the modified protein into cells is also a challenge (Siman and Brik, 2012).

GCE appears to be a promising approach for studying PTMs, as the incorporation of UAAs into proteins allows site-specific modification of PTMs and the generation of homogeneously modified proteins. The applications of UAAs in PTM research can be divided into two main categories: direct incorporation and indirect incorporation (incorporating precursors and converting them to the desired modifications in cells). Additionally, there have been studies that used photocaged UAAs or other methods to regulate the occurrence of PTMs by switching the activity of PTM-related enzymes (Mangubat-Medina and Ball, 2021). For example, a recent study incorporated photocaged lysine derivatives into *O*-GlcNAc transferase (OGT) to regulate the activation of OGT by photodecaging, enabling spatiotemporal precision control of cellular *O*-GlcNAc levels (He et al., 2022). This information will not be repeated here, and interested readers can refer to Section “*A switch for controlling protein activity*”.

Previous studies have shown that direct incorporation of UAAs in eukaryotic platforms can regulate PTMs such as Ser phosphorylation, Tyr sulfation, and Lys acetylation and acylation. However, for specific PTMs such as ubiquitylation that require UAAs with large modification groups (e.g., ubiquitylation, etc.), evolving the aaRS can be challenging. In addition, delivering ubiquitin-lysine into cells can also be challenging (Chen and Tsai, 2022). Therefore, researchers have used indirect incorporation methods, in which the precursor of the modified amino acid is incorporated into proteins and enzymatic reactions in cells are used to produce the protein with the desired modifications. This approach has been used to study the methylation, ubiquitination and ubiquitin-like modification of lysine and cysteine. In eukaryotic platforms, lysine-SUMOylation has been achieved using this method (Fottner et al., 2019). Table 3 summarizes the studies of modified UAAs in PTM research in eukaryotic systems.

**Table 3.** PTM modified UAAs in eukaryotic platform.

PTM	Amino acid	Structure	aaRS/tRNA	Eukaryotes	Action mode	References
Phosphorylation	Ser	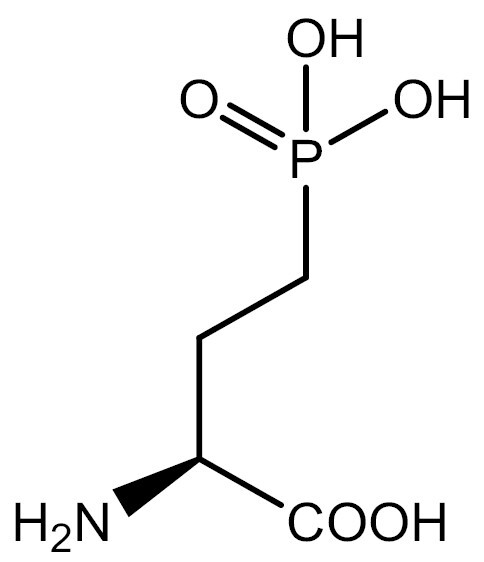	SepRS/tRNA	HEK293T	Direct incorporation	Beránek et al. (2018)
Sulfation	Tyr	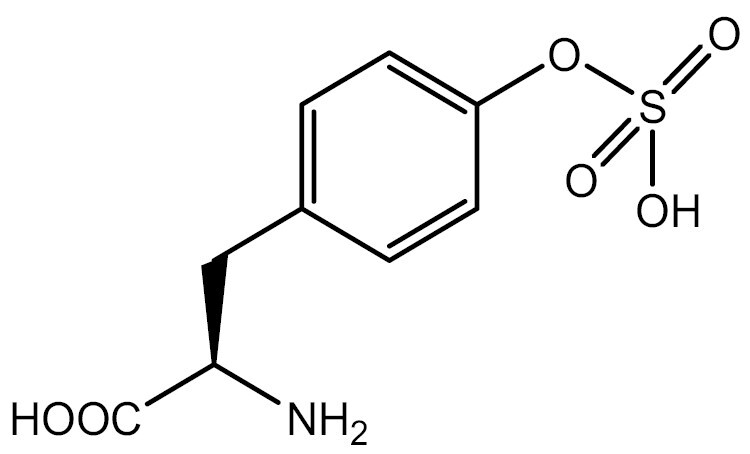	TyrRS/tRNA	HEK293T	Direct incorporation	Italia et al. (2020)
	Tyr		TyrRS/tRNA	HEK293T	Direct incorporation	He et al. (2020)
	Tyr		TyrRS/tRNA	HEK293T	Direct incorporation	Chen et al. (2022a)
Acetylation	Lys	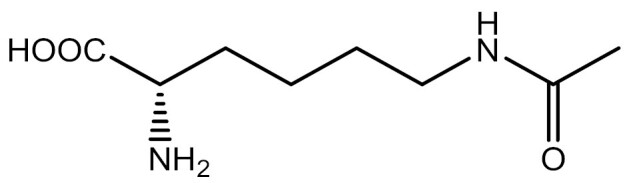	PylRS/tRNA	HEK293T	Direct incorporation	Mukai et al. (2008)
	Lys		PylRS/tRNA	Mouse embryonic stem cell	Direct incorporation	Elsässer et al. (2016)
Acylation	Lys	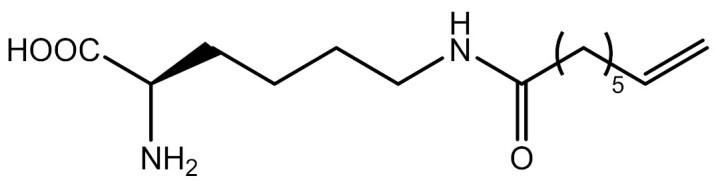	PylRS/tRNA	HEK293T	Direct incorporation	Wang et al. (2016b)
	Lys	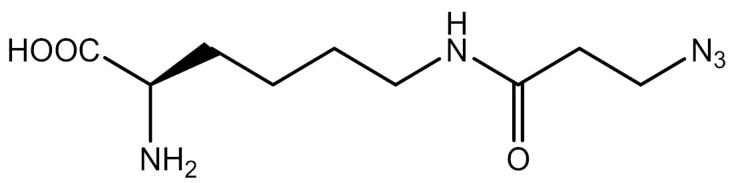	PylRS/tRNA	HEK293T	Direct incorporation	Wang et al. (2019)
SUMOylation	Lys	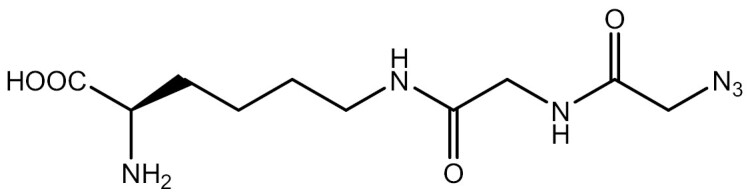	PylRS/tRNA	HEK293T	Indirect incorporation	Fottner et al. (2019)
Ubiquitination	Lys	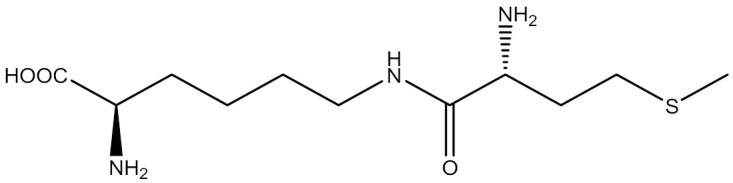 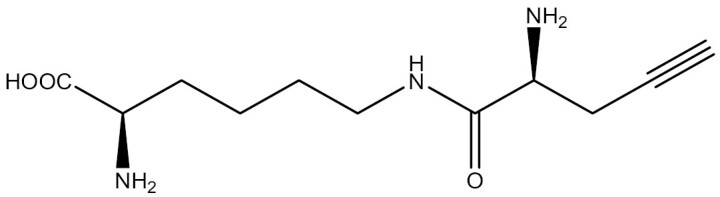	PylRS/tRNA	HEK293T	Indirect incorporation	Zang et al. (2023)

#### Protein interaction analysis

GCE-based photo- or chemical-crosslinking enables residue-selective crosslinking, making it a useful tool for studying protein interactions with other biomolecules. By incorporating UAAs carrying chemical groups with photocrosslinking or chemical-crosslinking abilities, the limitations of traditional chemical crosslinking methods, such as high false-positive identification rates, can be overcome. Additionally, this approach can capture weak and transient interaction partners, which may be missed by immunoprecipitation methods. In other areas, such as nanopores, which known for their high-throughput, cost-effectiveness, and rapid response as biosensors, the integration of UAAs can significantly enhance the chemical environment within the nanopore and increase its structural and functional diversity (Wu et al., 2023; Yang et al., 2023). Leveraging these advantages, these crosslinked UAAs not only serve as excellent tools for investigating protein interaction mechanisms, but also hold tremendous potential in antibody selection.

##### Photocrosslinking

To ensure the stability and functionality of photocrosslinked UAAs in cells, the photocrosslinking groups must be chemically stable and orthogonal to all the functional groups present in cells. Moreover, the excitation wavelength used for crosslinking should be a longer wavelength and not cause any harm to cells. These factors together can lead to a higher crosslinking efficiency and more stable complex formation.

**Figure 4.**
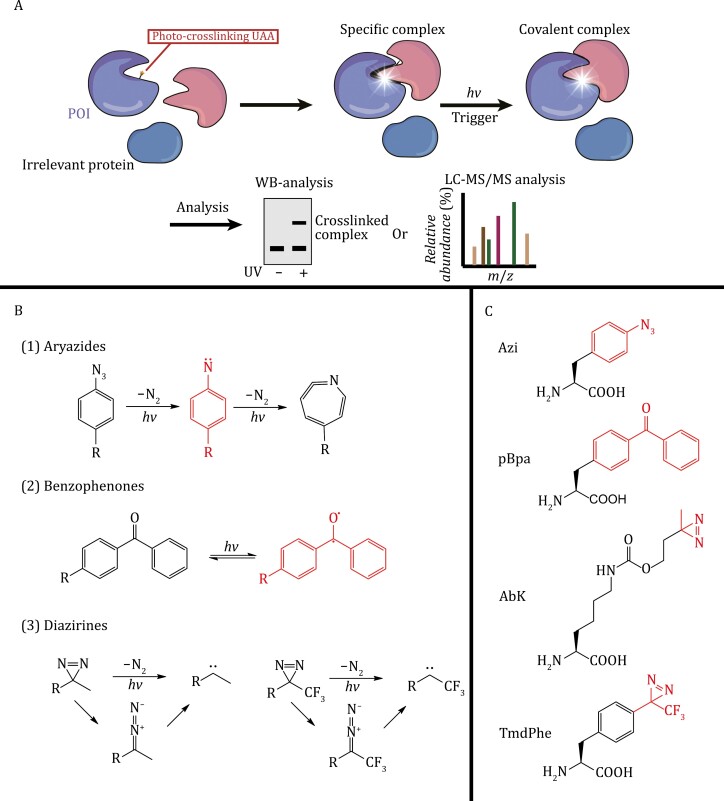
Schematic diagram of UAA photocrosslinking. (A) Mode of action for UAA photocrosslinking: upon incorporation of a UAA into the POI, the UAA can covalently bind to the target protein when exposed to specific wavelengths of irradiation. This method enables downstream analysis through techniques such as western blotting (WB) or liquid chromatography–mass spectrometry (LS–MS). (B) Activation mechanism of the three main photoreactive groups, including arylazides, benzophenone and diazirine. (C) Structures of four representative photocrosslinkable UAAs in eukaryotes.

Three major photoreactive groups, namely, aryl azides, benzophenone, and diazirine, have been incorporated into UAAs (Coin, 2018) (Fig. 4). Aryl azides are activated at a wavelength of 250 nm to form the reactive nitrene intermediate that inserts into nearby C–H and heteroatom–H bonds (Tanaka et al., 2008) [Fig. 4B (1)]. Benzophenones, which are activated at wavelengths of 350–365 nm, produce ketyl diradicals upon activation, showing a preference for γ–CH bonds (Coin, 2018) [Fig. 4B (2)]. Although benzophenones are widely used because of their high crosslinking efficiency, they have a bulky and rigid structure with a hydrophobic core and side reactivity with amines (Young et al., 2011). On the other hand, diazoxides, which are smaller in size than the previous two photocrosslinkers, are activated at wavelengths of 350–365 nm to produce a reactive carbene intermediate [Fig. 4B (3)]. The carbene intermediate readily inserts into C–H and heteroatom–H bonds (Coin, 2018). Diazoxides are characterized by rapid crosslinking, but the formation of diazo-isomers in the reaction can affect the efficiency of crosslinking. In a 2017 study, aryl azides and diazirines were found to be more effective than benzophenones in reducing off-target crosslinking and background noise (Kleiner et al., 2017). To function stably in cells, the photocrosslinking groups on UAAs need to be chemically stable and orthogonal to all the functional groups in cells. Additionally, the longer light wavelength used to excite the crosslinking activity should not be harmful to cells, thereby allowing a higher crosslinking efficiency and more stable complex formation.

In 2002, the successful incorporation of *p*-benzoyl-l-phenylalanine (pBpa), a photocrosslinkable UAA containing a benzophenone group, into the dimeric protein glutathione S-transferase in *E*. *coli* through the *Mj*TyrRS/tRNA pair, resulted in efficient crosslinking of protein subunits after irradiation. This work was the first application of UAAs for photocrosslinking control of proteins (Chin et al., 2002). However, since the *archaeal* TyrRS is not orthogonal in mammalian cells, Hino et al. (2011) introduced *Ec*TyrRS variants to achieve the incorporation of pBpa in mammalian cells. To further expand the specific synthetase available for photocrosslinkable UAAs in eukaryotic systems, Lacey et al. (2013) constructed highly diverse synthetase libraries and evolved *Mm*PylRS for pBpa and other phenylalanine derivatives with multiple conjugated aromatic rings to be incorporated in mammalian cells.

A significant portion of photocrosslinkable UAAs are derivatives of phenylalanine. These derivatives often have short, rigid side chains, and their photoactive groups are located close to the protein backbone, resulting in the formation of short covalent bridges that can capture only capture short-range interaction partners (Yanagisawa et al., 2012). This property limits the study of long-range protein crosslinking. To overcome this limitation, researchers have attempted to introduce lysine derivatives with photocrosslinkable groups using *Mb*PylRS/tRNA or *Mm*PylRS/tRNA pairs for long-range UAA photocrosslinking, resulting in the achievement of “wide-range” photocrosslinking of proteins in yeast and mammalian cells (Hancock et al., 2010; Yanagisawa et al., 2012).

Photocrosslinkable UAAs have been utilized to investigate various types of protein interactions, such as receptor–ligand interactions in cell signaling pathways, protein interactions in the chromatin, and protein interactions in living cells under different physiological conditions (Nguyen et al., 2018). Furthermore, photocrosslinkable UAAs play a significant role in antibody selection. In the early stages of antibody selection, preserving a broader sequence diversity is crucial for the development of therapeutic antibodies. Traditional phage display strategies often impose limitations on the diversity of identified antibody sequences and binding epitopes (Ledsgaard et al., 2022). By incorporating photocrosslinkable UAAs at antigen-specific sites, antibodies from the library can be readily crosslinked with UAAs, and subsequently the antibody binding to specific antigen epitopes can be screened. Currently, this technique was successfully implemented in *E*. *coli*, where Chen et al. (2020) incorporated pBpa into specific epitopes of IL-1β and complement 5a to select epitope-directed antibodies form phage display libraries. While there have been no reports of incorporating UAA into antigens in eukaryotic systems, the challenge is meaningful. After all, antigens expressed in eukaryotic systems undergo PTM, making them more capable of replicating the true interaction between antigens and antibodies *in vivo*. Figure 4C depicts some representative applications along with the photocrosslinkable UAAs used.

##### Chemical crosslinking and photoactivatable chemical crosslinking

**Figure 5.**
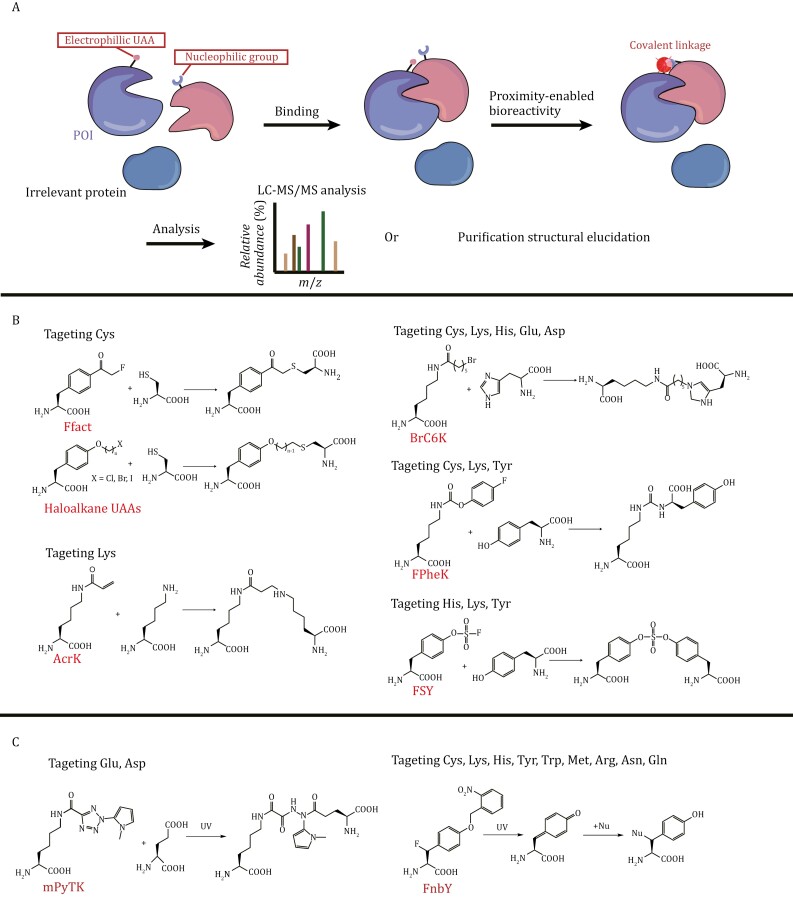
Schematic diagram of chemical-crosslinking of UAAs. (A) Mode of action for chemical-crosslinking of UAAs: UAAs containing electrophilic groups are incorporated into a POI and react with nearby amino acid residues containing nucleophilic groups, forming covalent bonds. (B) Reaction of chemical-crosslinking of UAAs with targeted AAs. (C) Reaction of photoactivatable chemical-crosslinking UAAs with targeted AAs.

The reaction of photocrosslinkable UAAs is characterized by the nonspecific insertion of reactive groups into C–H or heteroatom–H bonds of neighboring molecules after photoactivation. However, this reaction mode has a low yield as intermediates generated by the crosslinker are easily quenched by nucleophiles or water in the reaction environment, which can seriously affect the enrichment of interacting partners in subsequent studies (Coin, 2018). Therefore, a chemical-crosslinking modality with a different concept was proposed. The strategy to achieve chemical-crosslinking is to install an electrophilic group on the UAA, which will react with the nucleophilic proximal amino acid residues, such as the thiol group of Cys, to generate covalent linkages (Nguyen et al., 2018) (Fig. 5A). This mode of crosslinking does not require external triggering and provides residue specificity. Moreover, this chemical-crosslinking mode provides a higher crosslinking yield than photocrosslinking.

The electrophilicity of the UAA bearing a chemical crosslinker needs to be carefully adjusted to ensure that the reactive moiety has sufficient reactivity, high stability in cells, and high selectivity for the target amino acid residues (Liu et al., 2023). Currently, a variety of chemical crosslinkers have been installed on UAAs to achieve proximity reactions and target different residues (Nguyen et al., 2018). These representative structures and reactions are summarized in Fig. 5B.

Under physiological conditions, UAAs bearing chemical crosslinkers are unable to react with free natural amino acids, but when they are incorporated into target proteins, their crosslinked groups can react with adjacent natural amino acid residues. The first crosslinking reaction attempted was the reaction of halides with the thiol group of Cys to form a covalent crosslink. The first UAA used for chemical protein crosslinking was *p*-2ʹ-fluoroacetylphenylalanine (Ffact), which was designed in 2013 to introduce covalent bonds into proteins and was crosslinked with Cys, making an affibody irreversibly bind to its protein substrate, thereby achieving peptide–protein interaction capture in mammalian cells (Xiang et al., 2013). In addition to capturing and identifying protein–protein interactions, covalent crosslinking of UAAs helps stabilize low-affinity protein complexes on eukaryotic platforms (Cigler et al., 2017). Similar to the utilization of photocrosslinkable UAAs for antibody selection described earlier, chemically crosslinkable UAAs also have applications in antibody selection. Zhu et al. (2023) incorporated UAAs with crosslinking capabilities into antigen-specific epitopes to select antibodies produced by immunized mice that could form covalent crosslinks with the antigen. Nevertheless, the potential application of this technique in eukaryotic expression systems has yet to be investigated. Achieving covalent binding between proteins through chemical-crosslinking in eukaryotic systems is of great interest. Currently, this method has become an important strategy for the development of covalent protein drugs (Li et al., 2020; Wang and Wang, 2022; Yu et al., 2022). This topic will be covered in detail in Section “*Covalent protein drugs*”.

By combining photocrosslinking with chemical-crosslinking, a photoactivatable chemical crosslinker on a UAA has been developed. The photoactivated chemical group on the UAA releases a nonradical functional group under UV irradiation and selectively reacts with the targeted amino acid residue, thus improving the residue selectivity of photocrosslinking and the crosslinking efficiency, and avoiding potential harm from chemical-crosslinking agents in living systems. Tetrazole is a common photoreactive group, and after UAA photolysis, the nitrile imine is released from the tetrazole group and reacts with the nearby Glu residue to achieve protein crosslinking (Herner et al., 2016). For example, *N*-methylpyrroletetrazole lysine (mPyTK), a tetrazole-containing lysine derivative, was successfully incorporated into the growth factor receptor-bound protein 2 (Grb2) protein and crosslinked with epidermal growth factor receptor (EGFR) in mammalian cells (Tian et al., 2017) (Fig. 5C). To identify unknown protein interactions, crosslinkers that can target multiple amino acid residues have been developed. Quinone methide (QM), a photoactivated crosslinker that acts as a Michael acceptor for various nucleophiles (Toteva and Richard, 2011), can crosslink with 9 amino acid residues (Cys, His, Lys, Tyr, Trp, Asn, Gln, Asp, Glu, and Met). (2R)-2-Amino-3-fluoro3-(4-((2-nitrobenzyl)oxy) phenyl) propanoic acid (FnbY), which installs QM, has been developed and successfully incorporated into mammalian cells (Liu et al., 2019) (Fig. 5C).

#### Fluorescent labeling of proteins

Fluorescent labeling of proteins enables the accurate and even real-time detection of the protein conformation, interactions, and dynamics. Conventional fluorescent labeling methods include the fusion of a fluorescent protein (such as GFP) with the target protein or the addition of a fluorescent dye to react with the corresponding group of the protein. However, these methods are often not site-specific, and their development is limited by the size and photophysical properties of fluorescent proteins. The large size of a fluorescent protein can easily perturb protein function. Early attempts were made to achieve fluorescent labeling of proteins by modifying natural amino acids, such as methionine or alanine analogs, to be recognized by endogenous aaRS/tRNA pairs and react with fluorophores (Beatty et al., 2006; Dieterich et al., 2010). The disadvantage of this approach is that no specific protein can be tagged, and any endogenous methionine or alanine may be replaced by these modified amino acids.

**Figure 6.**
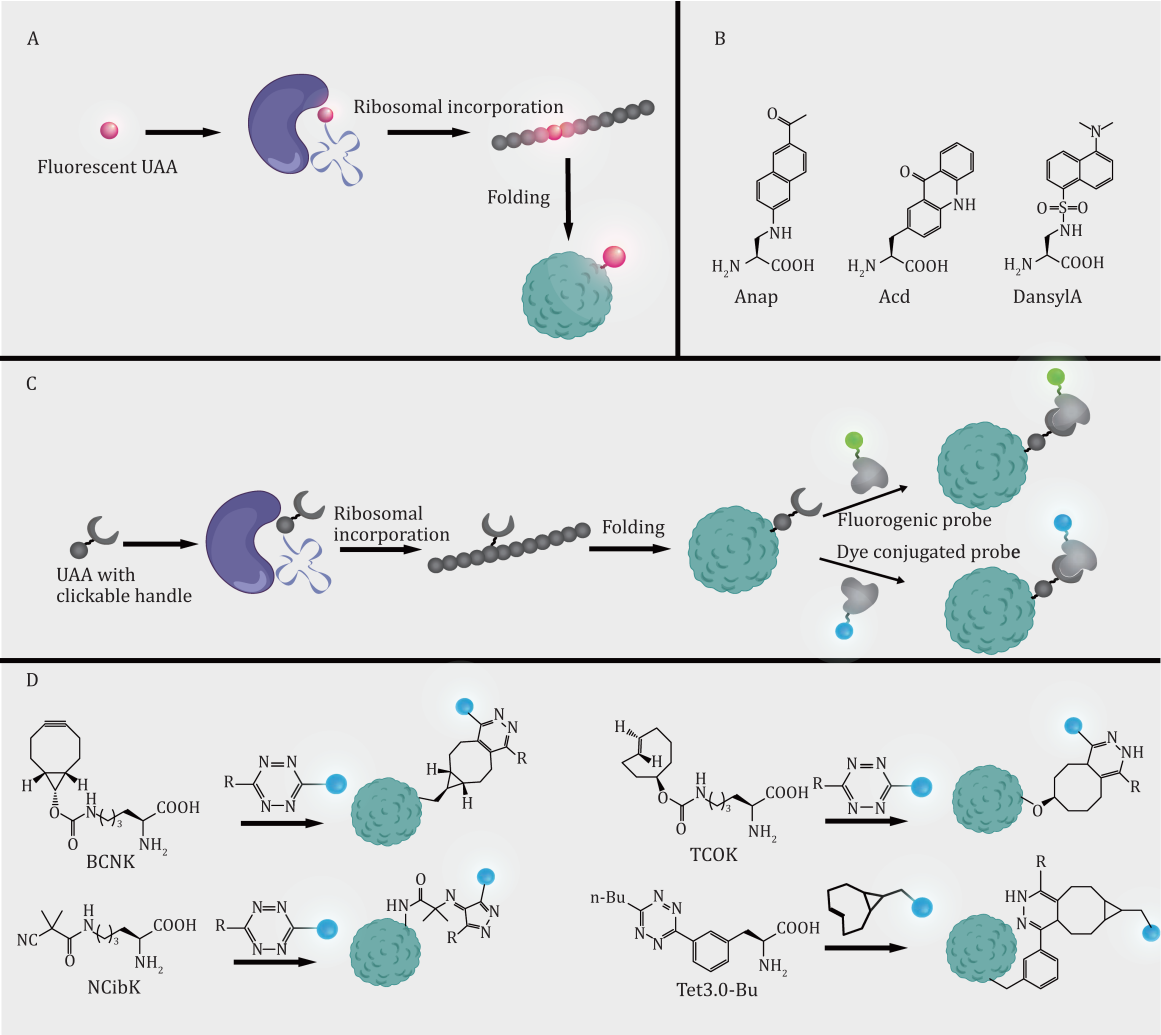
Reaction modes of genetic code expansion for fluorescently labeled proteins and the representative UAA structures. (A) Direct incorporation of fluorescent UAAs. UAAs with fluorophores are incorporated into the POI. (B) Representative structures of fluorescent UAAs commonly used in eukaryotes. (C) Site-specific UAA labeling using bioorthogonal chemistry: a UAA attached to an orthogonal handle is incorporated into the POI, followed by covalent binding to the orthogonal group in a fluorescent dye. (D) Representative structures of UAAs and reactions involved in site-specific UAA fluorescent labeling in eukaryotes.

The use of GCE facilitates the site-specific fluorescent labeling of a POI. There are two ways to fluorescently label proteins using GCE. The first method involves directly incorporating a UAA that has a fluorophore into the POI (Fig. 6A). However, UAAs with fluorophores are often bulky and inefficient. The second and commonly used method is to incorporate a UAA with an orthogonal handle into the POI using click reactions and covalently bind to orthogonal groups in the fluorescent dye (Fig. 6C and 6D). Both methods have been successfully applied to visualize proteins within eukaryotic systems.

Fluorescent amino acids (fAAs) enable site-specific introduction of fluorophores into POIs without the need for additional modification steps. One common fAA used for POI fluorescent labeling in mammalian cells is 3-((6-acetylnaphthalen-2-yl)amino)-2-aminopropanoic acid (Anap) (Fig. 6B). Anap is an amino acid analog of 6-propionyl-2-(*N*,*N*-dimethyl) aminonaphthalene (prodan), a highly environmentally sensitive fluorophore reported in previous studies. Anap inherits the environmentally sensitive nature of prodan, which makes it advantageous for studying protein dynamics. In 2009, Lee et al. (2009) synthesized Anap and efficiently incorporated it into *S. cerevisiae* using the *E*. *coli* LeuRS/tRNA pair. Recently, Anap has been used to study the subcellular localization of proteins (Chatterjee et al., 2013a), protein–protein interactions (Park et al., 2019, 2020) and membrane protein dynamics (Wulf and Pless, 2018; Shandell et al., 2019) in mammalian cells.

The site-specific fluorescent labeling of a POI achieved by a chemically orthogonal reaction between a UAA and fluorescent dye overcomes the shortcomings of fAAs in terms of the size, attachment position, and incorporation efficiency (Elia, 2021). A UAA with an orthogonal handle forms a covalent bond with a paired orthogonal group of a fluorescent dye though a click reaction, offering several advantages over fAAs. First, fluorescent dyes are smaller than fluorescent proteins, reducing the possibility of perturbing POI function and avoiding the restriction that fluorescent proteins can only attach to the N- or C-terminus of POI (Peng and Hang, 2016). Second, fluorescent dyes have excellent photophysical properties and are less phototoxic to cells than fluorescent proteins (Elia, 2021). Third, the click reaction is stoichiometric, with the fluorescent dye binding to a UAA in a 1:1 ratio (Serfling et al., 2019). This property facilitates the quantification of proteins by fluorescence intensity. Moreover, the slow incorporation reaction of a fAA limits its application, whereas click labeling between a UAA and fluorescent dye is rapid and facilitates quantitative site-specific protein labeling in a timely manner (Lang et al., 2012a).

In 2012, the Chin laboratory achieved rapid fluorescent labeling of a POI in mammalian cells by designing a lysine derivative loaded with norbornene through an inverse electron demand Diels–Alder cycloaddition reaction (IEDDA) with a tetrazine probe (Lang et al., 2012b). Further optimization was performed by designing a fast reaction of BCN- and *trans*-cyclooctene-containing amino acids with a tetrazine probe (Lang et al., 2012a). This reaction has the advantage of producing a single product of defined stereochemistry compared to the previous structure. These works laid the foundation for a subsequent series of bioorthogonal chemical labeling with small fluorophores on proteins. However, bioorthogonal chemical fluorescence labeling using tetrazine-based cycloaddition reactions mostly uses trained hydrophobic alkenes or alkynes, which limits their broad application. The Xiao laboratory has further developed UAAs containing small and hydrophilic isocyano or isonitrile groups for incorporation into mammalian cells. Through isonitrile–tetrazine coupling reactions, these methods provide good alternatives to hydrophobic alkanes or alkynethines (Chen et al., 2019) (Fig. 6D).

Based on the development and optimization of bioorthogonal chemical fluorescence labeling methods, GCE has found numerous applications for protein labeling in eukaryotic systems. This strategy has been successfully utilized for imaging various receptors and channels in living cells (Lang et al., 2012a; Nikić and Lemke, 2015; Gupta et al., 2019; Segal et al., 2020). Notably, this method has played a crucial role in super-resolution microscopy (Nikić et al., 2016) and single molecule labeling (Segal et al., 2020) techniques.

### Therapeutic applications

#### Protein drugs

##### Antibody-based drug delivery

The lack of specificity of chemotherapeutic drugs often leads to systemic side-effects. Antibody drug conjugates (ADCs) are a promising drug development strategy that has received considerable attention in recent years. A functional ADC consists of a highly selective humanized or human monoclonal antibody (mAb) that binds to a potent cytotoxic compound (payload) with a specific drug-antibody ratio (DAR) via a chemical linker (Leong et al., 2023). This approach combines the pharmacological potency of cytotoxic drugs with the specificity of therapeutic antibodies to develop biologic antibody therapies. ADCs have the advantage of delivering cytotoxic drugs to the tumor site with greater precision and efficiency, reducing the minimum effective dose and increasing the tolerated dose of the drug compared to conventional cytotoxic drugs (Schumacher et al., 2016).

Site-specific conjugation, as opposed to the conventional antibody conjugation method, avoids heterogeneous mixtures resulting from random reactions, which can significantly impact analytical characterization and manufacturing (Hao et al., 2021; Walsh et al., 2021). Additionally, site-specific conjugation greatly improves the homogeneity of the DAR, further reducing the ADC toxicity and enhancing its efficacy. GCE technology-produced ADCs are a representative example of third-generation ADCs for site-specific conjugation, which provides an efficient method for site-specific antibody modification, allowing the incorporation of UAAs into antibody sequences (Axup et al., 2012).

Recombinant antibodies for ADC preparation by GCE can be generated using either cell-based or cell-free systems (Walsh et al., 2021). While prokaryotic expression systems, such as *E*. *coli*, have high expression yields, they can only express simple proteins without glycosylation, which often fail to meet the requirements of antibody modification. Therefore, it is common to incorporate UAAs in mammalian cell platforms, particularly CHO cells, which ensures proper protein folding and PTM. It has been reported that the keto group of *para*-acetyl phenylalanine (pAcF) can be coupled with alkoxyamine-derived drugs to generate stable oxime bonds (Dirksen et al., 2006; Hutchins et al., 2011a, 2011b). Based on this principle, Axup et al. incorporated pAcF at specific positions in constant regions of trastuzumab (an anti-Her2 antibody) and coupled a C-terminal alkoxy-amine auristatin derivative with a modified trastuzumab mutant for the treatment of mammary fat pad tumors. These ADCs showed excellent antitumor efficiency in a xenograft model (Axup et al., 2012). In another study, researchers developed a CHO cell-based EuCODE expression system for the stable production of antibodies, incorporating UAAs with titers over 1 g/L. This expression platform was used to incorporate pAcF into anti-5T4 and anti-Her2 antibodies for site-specific coupling with the tubulin inhibitor monomethyl auristatin D (MMAD). The ADC developed using this expression platform showed a great efficacy (Tian et al., 2014). Additionally, Roy et al. developed a CHO cell-based UAA incorporation platform for producing UAA-containing ADCs. The ability of this platform to express UAA-containing IgG at high titers was demonstrated using perfusion bioreactors that allowed continuous addition of fresh media and nutrients and removal of cellular waste to maintain optimal growth and expression conditions. Furthermore, the researchers developed a scale-up process compatible with established upstream platform perfusion processes for future commercialization (Roy et al., 2020).

It is a common approach to construct ADCs by conjugating cytotoxic agents to antibodies that target tumor-associated antigens (TAAs). In addition, conjugating noncytotoxic agents such as kinase inhibitors or agonists to antibodies that target immune cells can help reduce off-target toxicity (Wang et al., 2015). The development of immunosuppressive ADCs has expanded the application of ADCs. Site-specific conjugation of drugs to antibodies helps improve the stability and pharmacokinetic properties of ADCs. Lim et al. incorporated pAcF into the heavy-chain constant region of anti-CD11a IgG in CHO cells. The pAcF on anti-CD11a IgG reacted with terminal aminooxy groups of liver X receptors (LXRs) agonists, resulting in LXR agonists targeting THP-1 monocytes/macrophages for treating atherosclerosis (Lim et al., 2015). Such agonists better target LXR on macrophages than traditional LXR agonists, contributing to the suppression of an array of inflammatory genes and inhibition of macrophage proliferation without activating LXR on hepatocytes, which has been reported to increase hepatic triglyceride levels (Lim et al., 2015).

In addition to delivering cytotoxic compounds, UAAs in antibodies can be utilized to deliver siRNAs or radioisotopes. Traditional tissue-specific siRNA delivery systems have drawbacks, including selective binding to tissue-associated antigens, which can result in siRNA degradation or off-target gene silencing. For example, antibody-protamine fusion proteins and scFv-9R conjugates have been used for selective tissue-specific siRNA delivery (Song et al., 2005; Kumar et al., 2008; Yao et al., 2012), but their effectiveness is limited. Similarly, nanostructures coated with targeted ligands also have drawbacks such as a poor binding affinity and low ligand density (Kim et al., 2008; Lee et al., 2012). To overcome these limitations, pAcF has been incorporated into an anti-Her2 Fab or the full-length IgG using *E*. *coli* or CHO expression systems, respectively, (Lu et al., 2013). The resulting antibodies can be conjugated to aminooxy-derivatized cationic block copolymers to generate antibody-polymer conjugates (APCs) with a more homogeneous structure and better affinity, resulting in low nonspecific cytotoxicity (Lu et al., 2013). These APCs were able to effectively target siRNA to Her2+ cells and silence genes at both the mRNA and protein levels.

Radioimmunotherapy is a therapeutic approach that involves labeling antibodies with radioisotopes using a bifunctional linker to specifically deliver the conjugate to cells expressing a specific antigen for imaging or treatment of lesions (Wu, 2009). Previously, isotopically labeled antibodies were generated by coupling bifunctional linkers with the thiol group of cysteine or the ε-amino group of lysine on mAbs. However, this approach resulted in heterogeneous products, which affected the activity of the conjugate (Adumeau et al., 2016). Wu et al. used a site-specific approach to introduce the UAA Nε-2-azideoethyloxycarbonyl-l-lysine (NEAK) into the anti-CD20 antibody rituximab using a 293F expression system. This modification allowed specific binding of the bifunctional linker 1,4,7,10-tetraazacyclotetradecane-1,4,7,10-tetraacetic acid (DOTA) to the antibody, and the rituximab conjugates were labeled with the radioisotope ^64^Cu or ^177^Lu (Wu et al., 2016). The conjugates produced in this way were highly homogeneous, and radiolabeling did not alter the binding affinity of rituximab to the antigens. This study provides a great idea for the development of radioimmunotherapy drugs.

##### Covalent protein drugs

Conventional targeted covalent inhibitors (TCIs) are small-molecule compounds that bind covalently to specific proteins, thereby inhibiting their biological functions. Covalent small molecule drugs typically contain functional groups, such as acrylamide, that can form covalent bonds with specific amino acid residues in targeted proteins (Baillie, 2016; Gehringer and Laufer, 2019). TCIs offer several advantages, including a prolonged drug efficacy and an increased therapeutic index. However, the off-target effects of covalent small molecules remain a major hurdle for their development (Singh et al., 2011; Sibaud et al., 2020).

In recent years, covalent protein drugs have garnered increasing attention owing to their higher specificity and greater potential for modification (Wang and Wang, 2022). However, achieving covalent binding between proteins has proven to be a challenge, primarily because proteins mainly interact through noncovalent bonds, and there are no naturally occurring amino acids that can form covalent bonds with one another (Darby and Creighton, 1995). Furthermore, amino acids are widely distributed in cells and cannot be selectively bound to target proteins (Wang and Wang, 2022). To address this problem, Xiang et al. proposed the concept of proximity-enabled bioreactivity (PERx) in 2013. This approach involves introducing potentially bioactive UAAs into proteins. UAAs can then react with proximal cysteine or lysine residues of targeted proteins, resulting in the formation of a new covalent bond that allows for irreversible binding of the utility-to-target proteins (Xiang et al., 2013). Initially, cysteine or lysine residues were used as suitable targets for covalent crosslinking, as the sulfhydryl group of cysteine is highly nucleophilic (Xiang et al., 2013), and lysine residues are relatively abundant on the protein surface and at interaction interfaces (Hacker et al., 2017). Based on these two targets of covalent crosslinking, a variety of UAAs have been developed and introduced into proteins for various applications, including increasing the thermostability of protein junctions (Xiang et al., 2014), performing specific optical modulation of protein structure and function (Hoppmann et al., 2014, 2015), and modifying antibodies to increase their stable binding to specific antigens on cancer cells (Furman et al., 2014).

The development of covalent protein drugs using PERx requires careful selection of the UAA, which must be chemically inert *in vivo* and able to efficiently react with neighboring target residues prior to drug-target dissociation. Additionally, it is essential to ensure that the UAA itself and its metabolic byproducts are noncytotoxic. Fluorosulfate-l-tyrosine (FSY) has been identified as a suitable UAA for the development of covalent protein drugs targeting programmed death-1 (PD-1) (Li et al., 2020). In this study, FSY was incorporated into PD-1 through PERx. When PD-1 interacted with PD-L1, FSY selectively reacted with a histidine residue near the contact site of programmed death ligand-1 (PD-L1). The resulting UAA-modified PD-1 was irreversibly bound to PD-L1, exhibiting potent antitumor activity (Li et al., 2020). Although FSY was integrated into PD-1 in *E*. *coli* in this study, FSY has the potential to be incorporated into proteins in eukaryotic systems. Wang et al. reported that FSY could also participate in proximity-enabled sulfur-fluoride exchange (SuFEx) reactions in mammalian cells, efficiently reacting with proximal lysine, histidine and tyrosine residues of target proteins. Therefore, FSY also holds the potential for incorporation into therapeutic proteins in eukaryotic platforms (Wang et al., 2018).

Previous studies have shown that FSY has great potential as an aryl fluorosulfate-containing UAA for the development of covalent protein drugs. However, because of its relatively rigid and short side chain, FSY has a relatively short reaction radius, which limits the diversity of proteins that can be targeted (Liu et al., 2021). To address this issue, Liu et al. developed a novel UAA, fluorosulfonyloxybenzoyl-l-lysine (FSK), which also contains an aryl fluorosulfate group (Liu et al., 2021). Studies have demonstrated that covalent nanobodies with incorporated FSY irreversibly bind to EGFR on cells. FSK and FSY target different sites on EGFR, counteracting the potential mutational resistance of EGFR. Researchers have also confirmed the protein-crosslinking ability of FSK in mammalian cells and demonstrated that FSK could be successfully incorporated into EGFP in HeLa cells and into proteins in 293T cells (Liu et al., 2021).

##### Bispecific antibodies

GCE has also been widely employed in the development of antibody drugs. This method allows the site-specific incorporation of UAAs with reactive groups, facilitating site-specific modification of antibody drugs and extending their application in immunotherapy. For reasons of speed and cost-effectiveness, the incorporation of UAAs into antibody fragments is typically achieved through bacterial expression systems, whereas incorporation into a full-length IgG is more dependent on eukaryotic hosts because of the requirement of PTM, such as glycosylation.

The development of bispecific antibodies (bsAbs) has addressed the issue of inefficacy in patients with low expression of target antigens (Lewis Phillips et al., 2008). As they bind not only to TAAs but also to an invariant component of T-cell receptors (TCRs) such as CD3 epsilon, bsAbs have been developed to enhance the efficacy of antibody drugs against tumors with low expression of Her2 (Rader, 2020).

Researchers have demonstrated that incorporating UAAs into specific sites of the Fab fragment of bsAbs can be beneficial for rational control of the binding site and the linker length between antibodies (Kim et al., 2012). To investigate the effects of different forms of bsAbs on cancer cells expressing different levels of Her2, Cao et al. designed four different structures of bsAbs. Using a CHO cell expression system, *para*-azidophenylalanine (*p*AzF) was incorporated into a specific site of the anti-Her2 IgG of trastuzumab and also into anti-CD3 Fab fragment using an *E*. *coli* expression system (Cao et al., 2015). BsAbs were developed based on IgG or Fab to bind either monovalent or bivalent CD3 Fab. In another study, the same approach was used to incorporate UAAs into specific sites of trastuzumab IgG in a CHO cell expression system for the production of bsAbs (Cao et al., 2021).

In addition to the CHO cell expression system, yeasts are one of the simplest and most cost-effective eukaryotic expression systems. *Pichia pastoris* is a promising host for recombinant protein production owing to its high secretion capacity and low levels of endogenous protein secretion (Choi et al., 2003). Additionally, *P. pastoris* is more amenable to high-cell-density cultivation than *S*. *cerevisiae*, leading to higher product titers (Young et al., 2009). Tir et al. employed P. pastoris to optimize the conditions for site-specific incorporation of the *p*AzF UAA into the trastuzumab Fab and full-length IgG. Through selection of appropriate fusion secretion signals and modulation of genes involved in protein folding and oxidative stress regulation, the authors were able to generate a full-length trastuzumab IgG with the *p*AzF modification in the light chain, with a titer of up to 15 mg/L. Furthermore, by knocking out YPT7 to prepare strains with disrupted vacuole sorting, they were able to improve the yield of *p*AzF incorporation into the trastuzumab Fab (Tir et al., 2022).

#### Cell-based therapies

##### Adoptive cellular therapy

With the advancement of designer cell-based therapies, researchers are exploring ways to treat diabetes by modifying β cells (Kitada et al., 2018) because the current treatment for patients with type 1 diabetes is still limited to blood glucose monitoring throughout the day and insulin injections. Small molecule and light-inducible therapeutic systems cannot accurately control blood glucose and achieve rapid insulin expression in diabetes treatment (Zaykov et al., 2016; Teixeira and Fussenegger, 2019). Researchers have developed engineered electrosensitive β-cells for the rapid release of insulin by implanting a specific bioelectronic device (Krawczyk et al., 2020). However, the regular maintenance of the bioelectronic device limits the wide application of this therapy. To achieve precise and rapid control of insulin expression, Chen et al. (2022b) developed a UAA-based therapeutic switch (NATS) system based on GCE. The NATS system is a modified mammalian cell that contains a bacterial aaRS/tRNA pair and an insulin gene carrying an ectopic amber codon. Microencapsulated cells can be implanted into mice, followed by oral intake of a UAA-containing inducer to achieve precise control of insulin expression. Diabetic model mice implanted with microencapsulated cells harboring the NATS system were able to achieve the relief of hyperglycemia within 90 minutes after the intake of the UAA inducer (Chen et al., 2022b). This method provides a new approach to diabetes treatment.

As a type of a living drug for cancer treatment, chimeric antigen receptor (CAR)-engineered T (CAR-T) cells have demonstrated a significant efficacy in the treatment of a variety of cancers. CAR-T-cell therapy enables T cells to recognize specific antigens on the surface of cancer cells by artificially modifying the T-cell receptor. CARs are hybrid receptors composed of an extracellular antigen-binding domain and an intracellular signaling domain (Rong et al., 2022). This structure helps T cells selectively target and eliminate cancer cells expressing the antigen of interest.

The activation of CAR-T cells needs to be precisely controlled, and the development of switchable CAR-T (sCAR-T) cells allows regulation of CAR-T-cell activity, to a certain extent. The use of GCE has been described in the development of sCAR-T cells (Ma et al., 2016). However, the efficacy of CAR-T-cell therapy against solid tumors is still insufficient (Maalej et al., 2023). In subsequent studies, researchers applied this method to develop sCAR-T cells targeting breast cancer cells with high Her2 expression; the developed sCAR-T cells showed a potent efficacy and safety, further expanding the application of CAR-T cells in the treatment of solid tumors (Cao et al., 2016). Rong et al. (2022) also reported the use of single-chain variable fragment (scFv) antibodies that incorporated the UAA pAcF to redirect dinitrobenzyl (DNP)-mediated universal CAR-T cells, which can improve the safety of CAR-T-cell therapy. However, because of technical bottlenecks, UAA incorporation in eukaryotic expression systems for sCAR-T-cell research still needs to be explored.

##### *In vivo* cell editing

Duchenne muscular dystrophy (DMD) is a disease caused by nonsense mutations resulting from deletions, duplications or point mutations in the DMD gene (Karijolich and Yu, 2014; Politano, 2021). The DMD gene encodes dystrophin, and the introduction of premature termination codons (PTCs) causes progressive muscle weakness and a shortened lifespan (Foster et al., 2012). Current treatments for nonsense mutation diseases include the use of aminoglycoside antibiotics or non-aminoglycoside drugs, which induce ribosomes to read through PTCs by randomly binding amino acids and produce full-length functional proteins (Asiful Islam et al., 2017). In addition to using PTC readthrough drugs, researchers have employed CRISPR-Cas9 genome editing to restore dystrophin (Long et al., 2014). Furthermore, Shi et al. (2022) applied the PylRS-tRNA^Pyl^ system to treat dystrophin expression disorders caused by point mutations in the DMD gene. In this study, the feasibility of using this UAA incorporation system to read through the endogenous PTC was verified in differentiated myoblasts derived from mdx mice with DMD point mutations and from DMD patients (Shi et al., 2022). Additionally, to deliver this UAA incorporation system *in vivo*, researchers constructed an AAV carrying this UAA incorporation system to provide a research basis for the clinical application of this method.

#### Vaccines

##### Protein-based vaccines

Vaccines developed for different types of diseases can be divided into two categories: protein-based vaccines and virus vaccines. The principle of protein-based vaccines is to elicit a strong immune response against specific epitopes or self-proteins that are weakly immunogenic or induce immunotolerance in patients (Fok and Mayer, 2020). Common strategies include nonselective chemical modification of endogenous proteins or introduction of chimeric antigens with foreign T-helper cell epitopes to elicit antibodies that are selective for both self-proteins and modified proteins and break immunotolerance (Weigle, 1965; Zuany-Amorim et al., 2004). However, these methods cannot precisely modify the target proteins, whereas GCE can achieve controllable and precise protein modification (Grünewald et al., 2009). In recent years, the GCE strategy has been applied to the development of protein-based vaccines for the prevention of autoimmune disorders or cancer.

For example, nitroaryl groups have been demonstrated to be highly immunogenic haptens, as they can attach to proteins and induce a strong immune response, which can help overcome autologous immune tolerance (Jones, 2015; Fok and Mayer, 2020). Therefore, proteins that are modified with immunogenic UAAs, such as *p*-sulfo-l-phenylalanine (SO_3_Tyr), 3-nitro-l-tyrosine(3NO2Tyr) or *p*-nitro-l-phenylalanine (*p*NO2Phe), when injected into the body, can produce high titer antibodies that are cross-reactive to the wild-type proteins (Grünewald et al., 2008). Vaccines, such as a recombinant RANKL vaccine, which utilizes site-specific incorporation of pNO2Phe into murine tumor necrosis factor-α (mTNF-α), have been developed for the prevention of collagen-induced arthritis and ovariectomy (OVX)-induced bone loss (Li et al., 2018; Wu et al., 2018b). However, the vast majority of these UAA incorporations were performed in prokaryotes. For the eukaryotic platform, DNP use has been reported for the incorporation of UAAs, which can be applied in HEK293T cells. Ren et al. (2015b) demonstrated that the DNP-containing UAA *N*(6)-(2-(2,4-dinitrophenyl)acetyl)lysine (DnpK) was unstable in *E. coli* but more stable in HEK293T cells and induced selective interaction with anti-DNP antibodies. This study suggests the possibility of future incorporation of DNP-containing UAAs in eukaryotic platforms for the development of protein-based vaccines.

##### Virus vaccines

Virus vaccines are generally classified into two types: live-attenuated virus (LAV) vaccines and inactivated whole virus particle vaccines. The latter are safer than the former, but require multiple booster shots to sustain the protection. LAVs typically elicit strong immune responses, but carry a risk of causing disease in immunocompromised individuals. The conventional approach to generating LAVs involves passaging the virus through an abnormal host or under non-physiological conditions to reduce its virulence and infectivity. However, both types of virus vaccines have limitations that hinder their wide application.

To address safety concerns associated with live-attenuated viruses, researchers have begun using molecular biology techniques to attenuate viruses in a more controlled manner. One such technique involves introducing PTCs into the viral genome, which results in incomplete translation of viral proteins and weakens the virus replication ability and virulence (Mayer, 2019; Wang et al., 2022). This approach was first applied to the HEK293T cell line in 2016 using GCE technology (Si et al., 2016). Researchers transferred an orthogonal translation system (*Mb*PylRS/tRNA_CUA_) into transgenic cells, inserted an amber stop codon into the genome of the influenza A virus, and analyzed mutations in multiple genes to produce PTC-harboring viruses. They observed the infectivity and stability of the progeny virus and found that PTC-harboring vaccines produced by this strategy exhibited strong immune effects on multiple organisms, providing a novel and versatile means for generating live virus vaccines (Si et al., 2016). Moreover, Yuan et al. (2017) designed an unnatural genetic switch using GCE technology in HEK293T cells to control the replication of live-attenuated human immunodeficiency virus type 1 (HIV-1) viruses. Based on the strategy of live virus vaccines using GCE technology, researchers have subsequently developed PTC-harboring vaccines for foot-and-mouth disease virus and pseudorabies virus (Hao et al., 2021; Wang et al., 2022).

## Prospectives

**Figure 7.**
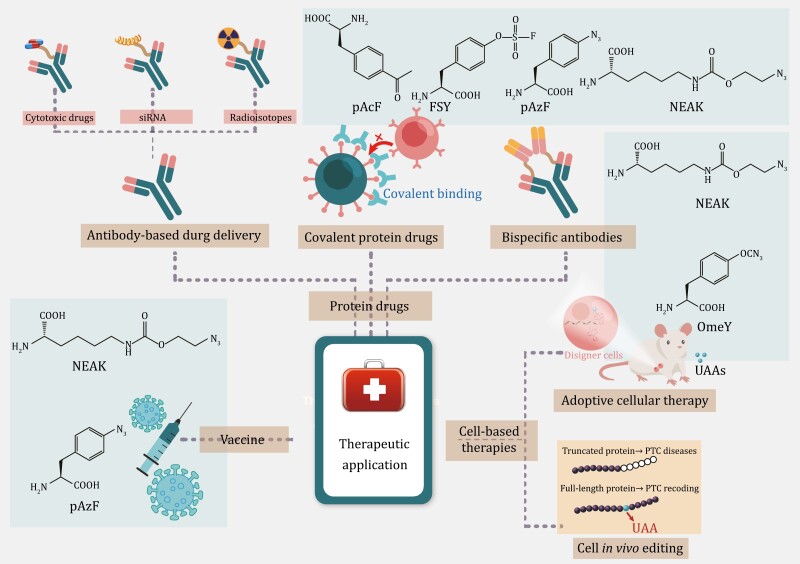
Application of genetic code expansion technology in eukaryotic expression systems for drug development and disease treatment, along with representative UAA structures.

GCE has become an attractive tool for modifying protein properties. Incorporating UAAs in eukaryotic platforms has significantly expanded the applications of GEC, allowing site-specific attachment of new chemical properties to target proteins, such as fluorescence, crosslinking or photocaging, to serve various research purposes. Currently, GCE is not limited to basic research and is gradually employed in the development of new disease treatment strategies (Fig. 7).

Although many bottlenecks have been addressed, GCE in eukaryotes still presents challenges. For instance, while efficient incorporation of UAAs has been achieved in some readily transfected cell lines, it remains challenging in other cell lines, with a low transfection efficiency of aaRS/tRNA pairs. This issue may necessitate further optimization of viral vectors and the suppression system. Moreover, researchers have focused on optimizing the structure and enhancing the incorporation efficiency of UAAs. Notably, release factor engineering has been reported to improve the UAA incorporation efficiency in mammalian cells. Additionally, the poor efficiency of heterologous tRNAs can be overcome through directed evolution of tRNAs in foreign host cells. However, this strategy has thus far only been implemented in bacterial expression systems and not in eukaryotic systems. Directed evolution of translation components (such as aaRS, tRNA, or EF-Tu) is typically a time-consuming task, and it allows the evolution of individual components in isolation rather than multiple components simultaneously. This limitation poses one of the most pressing challenges that needs to be addressed. Overall, with advancements in genetic manipulation, artificial intelligence, structural biology and interdisciplinary collaboration, the limitations of GCE technology encountered in eukaryotes will gradually be overcome, leading to further advancements in biotherapeutic applications.
